# A Novel Birthdate-Labeling Method Reveals Segregated Parallel Projections of Mitral and External Tufted Cells in the Main Olfactory System

**DOI:** 10.1523/ENEURO.0234-19.2019

**Published:** 2019-11-18

**Authors:** Tatsumi Hirata, Go Shioi, Takaya Abe, Hiroshi Kiyonari, Shigeki Kato, Kazuto Kobayashi, Kensaku Mori, Takahiko Kawasaki

**Affiliations:** 1 Brain Function Laboratory, National Institute of Genetics; 2 Graduate University for Advanced Studies, SOKENDAI, Mishima 411-8540, Japan; 3 Laboratory for Genetic Engineering, RIKEN Center for Biosystems Dynamics Research, Kobe 650-0047, Japan; 4 Laboratory for Animal Resource Development, RIKEN Center for Biosystems Dynamics Research, Kobe 650-0047, Japan; 5 Department of Molecular Genetics, Institute of Biomedical Sciences, Fukushima Medical University School of Medicine, Fukushima 960-1295, Japan; 6 Department of Physiology, Graduate School of Medicine, the University of Tokyo, Tokyo 113-0033, Japan

**Keywords:** birthdate tag, dopamine, olfactory system, olfactory tubercle, parallel pathway, tufted cell

## Abstract

A fundamental strategy in sensory coding is parallel processing, whereby unique, distinct features of sensation are computed and projected to the central target in the form of submodal maps. It remains unclear, however, whether such parallel processing strategy is employed in the main olfactory system, which codes the complex hierarchical odor and behavioral scenes. A potential scheme is that distinct subsets of projection neurons in the olfactory bulb (OB) form parallel projections to the targets. Taking advantage of the observation that the distinct projection neurons develop at different times, we developed a Cre-loxP-based method that allows for birthdate-specific labeling of cell bodies and their axon projections in mice. This birthdate tag analysis revealed that the mitral cells (MCs) born in an early developmental stage and the external tufted cells (TCs) born a few days later form segregated parallel projections. Specifically, the latter subset converges the axons onto only two small specific targets, one of which, located at the anterolateral edge of the olfactory tubercle (OT), excludes widespread MC projections. This target is made up of neurons that express dopamine D1 but not D2 receptor and corresponds to the most anterolateral isolation of the CAP compartments (aiCAP) that were defined previously. This finding of segregated projections suggests that olfactory sensing does indeed involve parallel processing of functionally distinct submodalities. Importantly, the birthdate tag method used here may pave the way for deciphering the functional meaning of these individual projection pathways in the future.

## Visual Abstract

**Figure:**
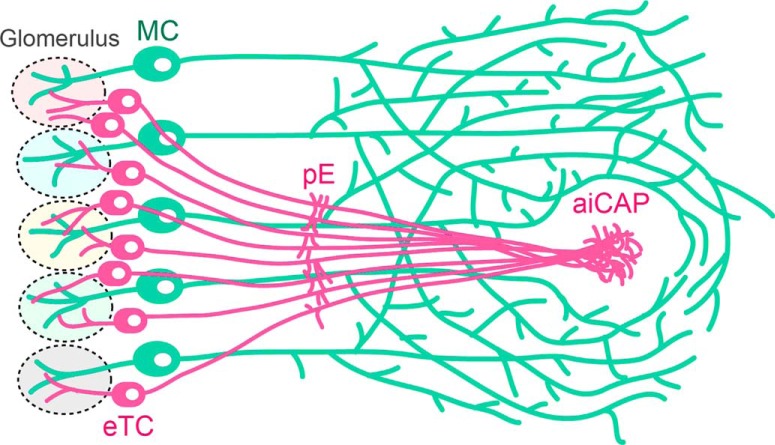

## Significance Statement

Odorant receptors form an ordered odorant map on the main olfactory bulb (MOB). This spatial representation disappears in most subsequent targets by “diffuse divergence and random convergence” of OB projections. We revisited these projections using a novel method that can genetically dissect distinct subsets of OB projection neurons based on their neuronal birthdates. Our birthdate tag analysis exposed parallel segregated projections formed by early-born mitral cells (MCs) and late-born external tufted cells (TCs) in otherwise apparently random olfactory networks. The results show that the birthdate tag approach is effective for uncovering a hidden circuit structure formed by intermingling neuronal subsets in the olfactory as well as other nervous systems.

## Introduction

Parallel processing is a fundamental strategy in sensory nervous systems (Young, 1998). In the visual system, for example, distinct submodalities such as color, orientation, or direction, are extracted from the visual scene by unique parallel computations and represented as individual higher-order maps in the central target (Nassi and Callaway, 2009). The olfactory system consists of two complete parallel pathways; the physically separated nasal and vomeronasal organs detect psychochemically distinct stimuli, which are in turn conveyed through segregated relay stations, the main olfactory bulb (MOB) and accessory olfactory bulb (AOB), to distinct sets of central targets (Dulac and Wagner, 2006). Within each pathway, a few subpathways are found peripherally between sensory neurons and the OB (Mori and Yoshihara, 1995; Brechbühl et al., 2008; Dewan et al., 2013; Greer et al., 2016). It remains unknown whether these subpathways extend parallel processing beyond the OB.

The MOB processes information of the complex odor scene through a large repertoire of odorant receptors. The nasal sensory neurons that express the same single odorant receptor converge their axons onto a few fixed glomeruli in the MOB, thereby representing the stereotypical odor map (Mori and Sakano, 2011). The projection neurons in the MOB then relay the output of single glomerulus to central target areas. Anterograde axon tracing of projection neurons assigned to the identified glomerulus revealed what appeared to be random, divergent projections of their axon terminals covering all of approximately ten target areas (Ghosh et al., 2011; Sosulski et al., 2011). Retrograde trans-synaptic tracing and postsynaptic electrophysiological recordings indicated that each central target neuron receives a combinatorial integration of outputs from multiple random glomeruli (Davison and Ehlers, 2011; Miyamichi et al., 2011). As a result, the MOB topography is undetectable other than in exceptional areas such as the pars externa (pE), a subdivision of the anterior olfactory nucleus (AON; Schoenfeld and Macrides, 1984; Yan et al., 2008) and parts of the amygdala, in which dorsal or ventral glomerular output is overrepresented (Miyamichi et al., 2011; Inokuchi et al., 2017). Instead, olfactory information processing is generally characterized by diffuse divergence from a single glomerulus to all the target areas and random convergence from multiple glomeruli to each target neuron (Wilson and Sullivan, 2011), making it challenging to deduce the logic underlying the information processing.

A critical question in understanding the olfactory logic relates to the connection specificity of projection neuron subtypes. The projection neurons are classified into mitral cells (MCs) and tufted cells (TCs) based on the location of their cell bodies in MOB layers. The information of each glomerulus is relayed by ∼20 MCs and ∼50 TCs to the central targets (Nagayama et al., 2014). TCs are a heterogeneous group, and further subclassified into internal, middle TCs (imTCs) and external TCs (eTCs) subtypes by their locations within the external plexiform layer (EPL) and the glomerular layer (GLL). These projection neuron subtypes have distinct intrabulbar connections (Mori et al., 1983; Orona et al., 1984) and electrophysiological properties (Nagayama et al., 2004; Griff et al., 2008a; Fukunaga et al., 2012), suggesting that they could carry different kinds of information. A few studies have reported that TCs project only to the anterior olfactory targets involving the AON, olfactory tubercle (OT), and anterior piriform cortex (PC; Haberly and Price, 1977; Scott, 1981; Nagayama et al., 2010). In particular, single cell dye labeling has revealed that the terminal branches of several imTCs accumulate around the boundary between the OT and anterior PC (Igarashi et al., 2012). However, since there are no good molecular markers to distinguish the MOB projection neurons, it remains unclear whether these heterogeneous populations simply make a convergent map or segregated maps in the olfactory target areas.

A potential approach to dissect the OB projection neurons is to make use of differences in their birthdates. Neuronal birthdating using nucleotide analogs is a well-established standard for the classification of neuronal subtypes (Bayer and Altman, 1987). It has led to many important discoveries while undergoing very little technological improvement. The obvious limitation is that it remains an entirely histologic method, capable only of visualizing cell nuclei that have incorporated the analogs in fixed samples.

This study developed a novel birthdating method by which Cre-loxP recombination is induced in a neuronal birthdate-dependent manner. Using this method, we demonstrate the segregation of axon trajectories of OB projection neuron subsets into parallel projections. Specifically, a subset of eTCs birthdate tagged at embryonic day (E)15.5 projected to only two small targets deviated from other projections. Their selective target in the OT was then analyzed in more details.

## Materials and Methods

### Mouse breeding and experiments

All animal procedures were performed in accordance with the animal care committee's regulations at the National Institute of Genetics and RIKEN Center for Biosystems Dynamics Research (BDR). Although both male and female mice were used for generation of experimental materials, datasets were obtained from only male mice after their sexes were externally apparent around one week of age. The sexes of younger mice were undetermined, but it is highly likely that only male mice were used for sampling because the transgene of the birthdate tag driver Nerog2^CreER^(G2A) seems to be located in the Y chromosome.

The day on which a vaginal plug was detected was designated as E0.5, and the day of birth was designated as postnatal day (P)0. Tamoxifen (TM) treatment was performed by intraperitoneally injecting 250 μl of corn oil (Sigma-Aldrich catalog #C8267) containing 9 mM TM (Sigma-Aldrich catalog #T5648) and 5 mM progesterone (Wako catalog #161-14531) into a staged pregnant mouse. Because TM often delays delivery, when pups were not born by E19.5, they were collected by caesarian delivery and given to ICR foster mothers.

### Mouse lines

The mouse lines used in this study are listed in Table 1. The birthdate tag driver line Neurog2^CreER^(G2A) (accession number CDB0512T−1: http://www2.clst.riken.jp/arg/TG%20mutant%20mice%20list.html) was established using a genomic BAC clone RP23-360O22 encoding the mouse neurogenin (Neurog)2 gene obtained from the BACPAC Resource Center (Children’s Hospital Oakland Research Institute, Oakland, CA). The entire coding sequence in exon2 was replaced by a TM-inducible Cre recombinase, CreER^T2^ (Feil et al., 1997; a generous gift from Dr. Pierre Chambon, University of Strasbourg) through homologous recombination as described elsewhere (Oginuma et al., 2008). The BAC recombinant was injected into fertilized eggs with a C57BL/6 background, and the resulting mice were assayed for integration of the transgene by PCR of the genomic DNA. The three mouse lines that independently received the transgene integration were then assayed for the Cre recombinase activities by crossing with ROSA26R Cre reporter mice (Soriano, 1999). For the assay, the pregnant mice were injected with TM solution at E12.5 or E14.5, and brains of the embryos were dissected out at E18.5–E19.5 and whole-mount stained with X-gal (5-bromo-4-chloro-3-indoyl-β-D-galactopyranoside) as described elsewhere (Saga et al., 1992). Through this screen, the birthdate tag line Neurog2^CreER^(G2A) was selected by the highest recombination rate in OB neurons. Transgene containment was routinely determined with PCR using internal Cre recombinase primers, 5′-TAAAGATATCTCACGTACTGACGGTG-3′ and 5′-TCTCTGACCAGAGTCATCCTTAGC-3′, resulting in amplification of 300-bp fragments.

**Table 1.** List of mouse lines used in this study

Mouse line	Genomic locus	Enhancer/promoter	Reporter/effector	Source	Description
Neurog2^CreER^(G2A)	Unknown transgenic insertion	Neurog2	Cre-ER	This article #CDB0512T−1	Neuronal birthdate tag driver designed for TM administration to induce recombination of loxP sequences in a neuronal birthdate-dependent manner
Tau^mGFP-nLacZ^	Mapt (microtubule-associated protein tau) locus	Mapt:widespread neuronal expression	Membrane-bound GFP and nuclear-targeted β-gal	Hippenmeyer et al. (2005) JAX stock #021162	After Cre-loxP recombination, dual reporter proteins are expressed in neurons throughout the nervous system
Cdhr1^tTA^	Unknown transgenic insertion	Cdhr1: OB-specific expression	tTA	This article #CDB0535T:	After Cre-loxP recombination, tTA isexpressed in OB projection neurons
TRE^tdTomato-sypGFP^	Unknown transgenic insertion	TRE	tdTomato and synaptophysin-fused GFP	Li et al. (2010) JAX stock #12345	Designed for tTA to drive ubiquitous expression of dual reporter proteins in any types of cells
ROSA26-TRE^mGFP^	ROSA26 locus	TRE	Membrane-bound GFP	This article #CDB0300K	Designed for tTA to drive ubiquitous expression of the reporter protein in any types of cells

The Cdhr1^tTA^ mouse line (accession number CDB0535T: http://www2.clst.riken.jp/arg/TG%20mutant%20mice%20list.html) was established using a mouse genomic BAC clone RP23-55P20 (BACPAC Resource Center), which encodes cadherin related family member (Cdhr)1 gene that exhibits OB-specific expression (Nagai et al., 2005). At 25 bp upstream of the translation initiation site of this gene, a loxP-LacZ-loxP-tTA cassette was inserted through homologous recombination. In this cassette, the LacZ gene followed by a transcription termination site was flanked by loxP sequences and used as a STOP sequence, which followed by the tetracycline-controlled transactivator (tTA) gene in an optimized form for mammalian expression (Inamura et al., 2012; a generous gift from Dr. Kazuhiro Ikenaka, National Institute for Physiologic Sciences). The original loxP site that had existed in the vector backbone (pBACe3.6) was deleted by replacement using p23loxZeo (a generous gift from Dr. Junji Takeda, Osaka University). The constructed recombinant BAC was then injected into fertilized eggs with a C57BL/6 background. Among the six independent transgenic lines obtained, the Cdhr1^tTA^ was characterized by the OB-specific strongest expression of the internal LacZ gene. The Cdhr1^tTA^ transgene integration was routinely determined with PCR using internal LacZ primers, 5′-CATTGGCGTAAGTGAAGCGAC-3′ and 5′-ATCCCAGCGGTCAAAACAGG-3′, resulting in amplification of 381-bp fragments

The ROSA26-TRE^mGFP^ (accession number CDB0300K: http://www2.clst.riken.jp/arg/TG%20mutant%20mice%20list.html) was developed as reported previously (Abe et al., 2011). The tetracycline response element (TRE) cloned from pTRE tight2 (Addgene plasmid # 19407, a generous gift from Dr. Markus Ralser, University of Cambridge) was connected to a GFP gene containing a palmitoylation sequence of GAP43 N terminally, and the construct was flanked by 2xcHS4 insulators (Stewart et al., 2009). To generate a targeting vector, the 2xcHS4 TRE-mGFP with an frt-flanked neomycin resistant gene was inserted into the pMC1-DT-A-ROSA26 vector (Abe et al., 2011). The targeting vector was introduced into the HK3i embryonic stem cells derived from C57BL/6 (Kiyonari et al., 2010). After screening by PCR and Southern blotting, the targeted ES clones obtained were injected into ICR 8-cell stage embryos to generate chimeric mice, after which the chimeric mice were mated with Tg(ACTFLPe)9205Dym transgenic mice (Rodríguez et al., 2000) to remove the neomycin cassette. The offspring were genotyped with PCR using the primers, 5′-GCGATCACATGGTCCTGC-3′ and 5′-AATCGGCCGCTCTAGAAC-3′, for 305-bp fragments from the knock-in mutant allele.

The Tau^mGFP-nLacZ^ mice (Hippenmeyer et al., 2005; JAX stock #021162, The Jackson Laboratory) with a CD-1 mixed background were provided by Dr. Silvia Arber (Friedrich Miescher Institute for Biomedical Research), and backcrossed with C57BL/6 wild-type mice for at least four generations before use in this study. The TRE^tdTomato-sypGFP^ mice (Li et al., 2010; JAX stock #12345) with the FVB background were obtained from the Jackson laboratory and backcrossed with C57BL/6 mice at least three generations before use in this study.

### Histochemistry

**Figure 1.**
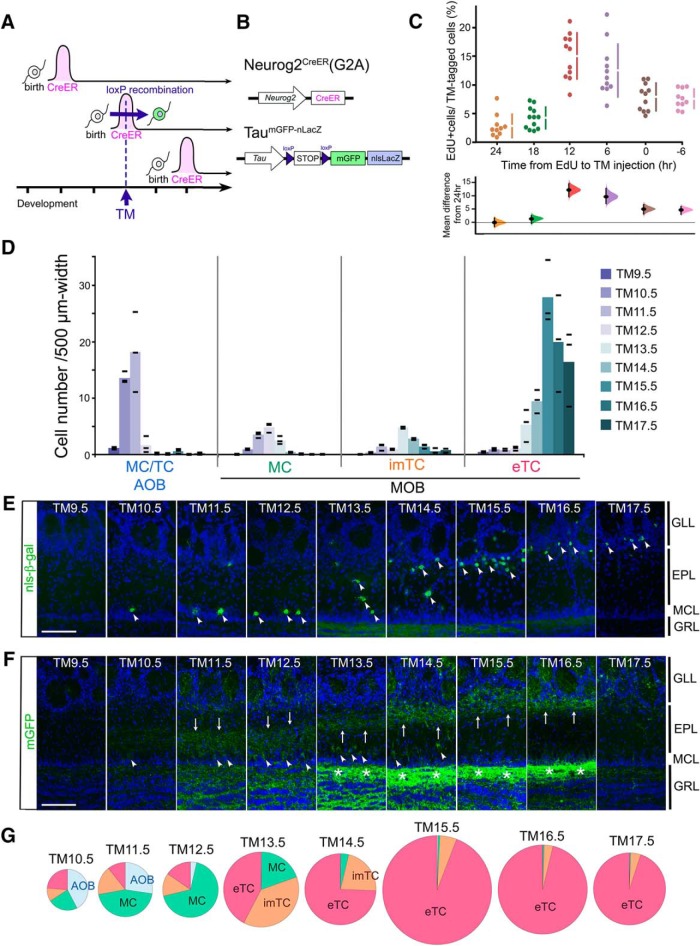
Birthdate tagging of OB projection neurons. ***A***, The experimental design of the birthdate tag method. The horizontal axis indicates the developmental time course. Individual neurons express CreER only transiently, soon after birth. An injection of TM at a certain developmental time point induces Cre-loxP recombination only in neurons expressing CreER. ***B***, Schematized gene structures of the birthdate tag driver Neurog2^CreER^(G2A) and Tau^mGFP-nLacZ^ reporter used in this figure. ***C***, Proportion of EdU-incorporated cells in birthdate-tagged neurons shown by Cumming estimation plot. TM was given at a fixed time point at E12.5, and EdU was given at the indicated time before the TM injection. The raw values calculated from individual mice and mean ± SD are plotted on the upper axis. On the lower axis, mean differences from the 24-h group are plotted as bootstrap sampling distributions. Each mean difference is depicted as a dot. Each 95% confidence interval is indicated by the ends of the vertical error bars. ***D***, The number of birthdate-tagged neuron subtypes counted in OB sections prepared from P14 Neurog2^CreER^(G2A); Tau^mGFP-nLacZ^ mice that were given TM at different embryonic stages. The cell numbers are normalized by the layer distance used for cell counting. The color intensity coded columns show the means of tagged-cell numbers at individual TM injection stages. Black bars show the raw values obtained from individual mice (*n* = 3 for each TM stage). ***E***, ***F***, Immunostaining of MOB sections prepared from P14.5 Neurog2^CreER^(G2A); Tau^mGFP-nLacZ^ mice for nucleus-localized β-gal (***E***) and membrane-bound GFP (***F***) reporters. TM was administrated at different embryonic stages as indicated at the top. Arrowheads (***E***, ***F***) indicate reporter-tagged cell bodies, and arrows (***F***) indicate the position of reporter-tagged dendrites in the EPL. Asterisks (***F***) show the reporter-labeled internal plexiform layer that contains intrabulbar association fibers. Scale bar = 100 μm. GRL: granule cell layer. ***G***, Proportion of the neuron subtypes birthdate tagged at different TM stages in the whole OB. The proportions were calculated without normalization by the tissue size, and therefore AOB neurons may look underrepresented compared with ***D***, reflecting the small size of AOB. The diameter of each pie chart is proportional to the number of neurons. The exact numbers of neurons counted are shown in Extended Data Figure 1-1.

**Figure 2.**
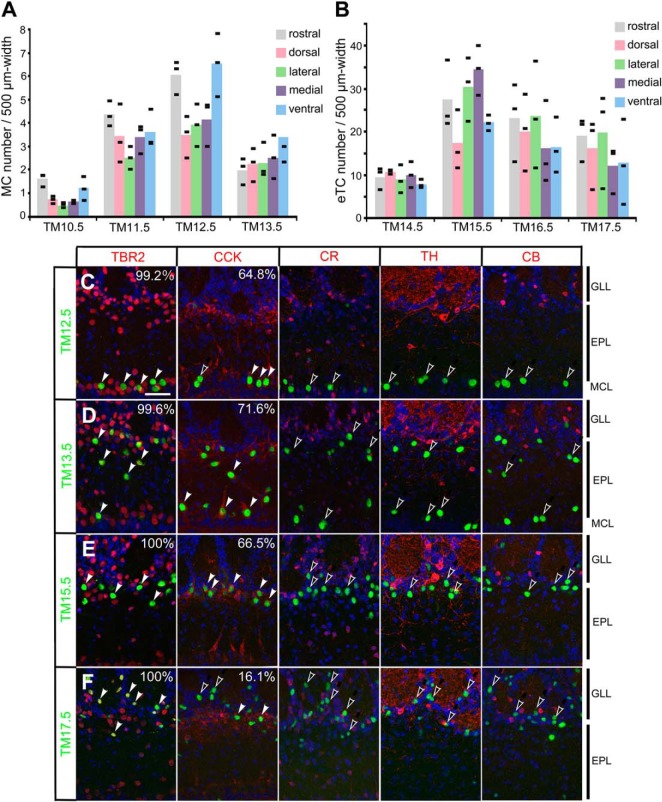
Characterization of birthdate-tagged neurons in the MOB. ***A***, ***B***, Distribution of birthdate-tagged MCs (***A***) and eTCs (***B***) in different OB sectors. The numbers of MCs and eTCs are counted from the rostral, dorsal, lateral, medial and ventral sectors of MOBs prepared from P14 Neurog2^CreER^(G2A); Tau^mGFP-nLacZ^ mice that were given TM injection at the embryonic stages indicated at the bottom. Black bars indicate the raw values obtained from individual mice (*n* = 3 for each category), and colored columns represent the means. ***C–F***, Expression of neuron subtype markers in β-gal reporter-labeled (green) neurons that were tagged at TM12.5 (***C***), TM13.5 (***D***), TM15.5 (***E***), and TM17.5 (***F***) in P14.5 Neurog2^CreER^(G2A); Tau^mGFP-nLacZ^ MOBs. Red staining shows TBR2 (projection neuron marker), cholecystokinin (projection neuron marker), calretinin (periglomerular cell marker), tyrosine hydroxylase (interneuron marker) and calbindin (periglomerular cell marker) from left to right. DAPI (blue) staining is superimposed in all panels. The filled white arrowheads indicate marker-positive birthdate-tagged neurons, and the open arrowheads indicate marker-negative birthdate-tagged neurons. The percentage of marker-positive in β-gal-positive neurons is shown on the right top corner in each panel. The percentage is not shown when no marker-positive cells were found in β-gal-positive birthdate-tagged neurons. The positions of MOB layers are indicated on the right-hand end. Scale bar = 50 μm.

Animals were anesthetized and transcardially perfused with 4% paraformaldehyde (PFA)/PBS. The brains were dissected and further fixed with 4% PFA/PBS for 12–24 h, immersed in 30% sucrose/PBS, and frozen in the mixed solution of OCT-compound (Sakura Finetek) and 30% sucrose/PBS at a ratio of 2:1. Coronal sections with thickness depending on mouse age (16 μm for E19.5–P0, 20 μm for P2–P16, 30 μm for P20–P23, unless otherwise indicated) were cut on a cryostat, placed on MAS-coated glass slides (Matsunami Glass), and immunostained as described previously (Mita et al., 2015). The primary antibodies used were as follows: rabbit anti-β-gal (1:5000, Invitrogen catalog #A11132, an old lot); chicken anti-β-gal (1:2000, Abcam catalog #ab9361, RRID: AB_307210); rabbit anti-GFP (1:1000, #598, MBL International catalog #598, RRID: AB_591816); rabbit anti-TBR2 (1:1000, Abcam catalog #ab23345, RRID: AB_778267); rabbit anti-cholecystokinin 26-33 (1:500, Sigma-Aldrich catalog #C2581, RRID: AB_258806); rabbit anti-calretinin (1:5000, Millipore catalog #AB5054, RRID: AB_2068506), rabbit anti-tyrosine hydroxylase (1:1000, Millipore catalog #AB152, RRID: AB_390204); rabbit anti-calbindin D-28K (1:1000, Millipore catalog #AB1778, RRID: AB_2068336); rabbit anti-GFP (1:1000, #598, MBL International catalog #598, RRID: AB_591816); rabbit anti-DARPP-32 (1:1000, Abcam catalog #ab40801, RRID: AB_731843); goat anti-dopamine receptor 1 (1:1000, Frontier Institute catalog #D1R-Go-Af1000, RRID: AB_2571594); rabbit anti-dopamine receptor 2 (1:1000, Frontier Institute catalog #D2R-Rb-Af960, RRID: AB_2571596); and mouse anti-GAD67 (1:300, Millipore catalog #MAB5406, RRID: AB_2278725) antibodies. Chicken instead of rabbit anti-β-gal antibody was used in double staining double staining with with EdU (Fig. 1*C*) and with rabbit primary antibodies (Fig. 2*C–F*). In the immunostaining shown in Figures 2*C–F*, 11*D*, sections were autoclaved at 105°C for 2 min in an antigen retrieval solution (HistoVT One, Nakalai Tesque) before the primary antibody reactions. The secondary antibodies used were donkey Alexa Fluor 488-conjugated anti-rabbit IgG (1:1000, Life Technologies catalog #A-21206, RRID: AB_141708), donkey Cy3-conjugated anti-rabbit IgG (1:1000, Jackson ImmunoResearch catalog #711-165-152, RRID: AB_2307443), donkey Alexa Fluor 647-conjugated anti-rabbit IgG (1:1000, Jackson ImmunoResearch catalog #711-605-152, RRID: AB_2492288), donkey Cy3-conjugated anti-mouse IgG (1:1000, Jackson ImmunoResearch catalog #715-165-150, RRID: AB_2340813), donkey Alexa Fluor 488-conjugated anti-chicken IgY (1:1000, Jackson ImmunoResearch catalog #703-545-155, RRID: AB_2340375), donkey Alexa Fluor 488-conjugated anti-goat IgG (1:1000, Life Technologies catalog #A-11055, RRID: AB_2534102), and donkey Alexa Fluor 647-conjugated anti-goat IgG (1:1000, Jackson ImmunoResearch catalog #705-605-147, RRID: AB_2340437) antibodies. Some sections were counterstained with DAPI (4',6-diamidino-2-phenylindole; Wako catalog #045-30361) and FluoroMyelin RED (Invitrogen catalog #F34652) according to the manufacturer's instructions.

10.1523/ENEURO.0234-19.2019.f1-1 Extended Data Figure 1-1 Number of neurons counted in Fig.1. Download Figure 1-1, TIF file.

### Image acquisition and analysis

The fluorescent images were captured with an inverted confocal microscope (Olympus IX81 FV1000) through 10×, 20×, and 40× objectives (UPlanSAPO) using Fluoview (FV-ASW ver4.02) software and automatically pseudo-colored. Wide images were automatically constructed via multi-area time-lapse with the built-in mosaic-imaging tool. Only the fluorescent images in Figure 1*E* were taken with a CCD camera (Olympus DP71) on a conventional fluorescent microscope (Zeiss Axioplan2) through a 20× objective lens (FLUAR) using Cell Sens Standard software. The obtained images were rotated and cropped, and the brightness and contrast were non-linearly adjusted equally across of the entire image using Photoshop CS5 software (Adobe).

### EdU incorporation assay

A thymidine analog, 5-ethynyl-2'-deoxyuridine (EdU; Tokyo Chemical Industry catalog #E1057) was intraperitoneally injected into a pregnant mouse (50 mg/kg body weight) at the indicated time point either before or after the TM injection that was given at the fixed E12.5. Brains were dissected from the embryos at E19.5 and cryosectioned as explained above. The sections were antigen-retrieved as described above and immunostained with chicken anti-β-gal and the secondary antibody first. Subsequently, the incorporated EdU was detected in 0.1 M Tris (pH 7.6), 2 mM CuSO_4_, 3 μM Alexa Fluor 555 azide (Thermo Fisher Scientific catalog #A20012), and 10 mM ascorbic acid for 40 min at room temperature. The numbers of neurons labeled for β-gal and those doubly labeled for β-gal and EdU were manually counted in eight independent visual fields (containing 127-307 β-gal-positive neurons) in MOB sections for each mouse with a 40× objective lens (Plan-Apochromat) under a fluorescent microscope (Zeiss Axioplan2) using filter sets 38 HE (ex470/40, em525/50) and 15 (ex549/12, em590). Because the plug-based staging was adopted in this study, the exact time of conception was uncertain, resulting in interlitter variation of development of up to 12 h (Heimer et al., 1987). Therefore, to reduce the litter bias, at least four different litters were used for each quantification; –6 h: 10 mice from four litters; 0 h: 11 mice from five litters; 6 h: 11 mice from four litters; 12 h: 11 mice from four litters; 18 h: 12 mice from four litters; 24 h: 10 mice from four litters.

### Quantification of birthdate-tagged OB neurons

**Figure 3.**
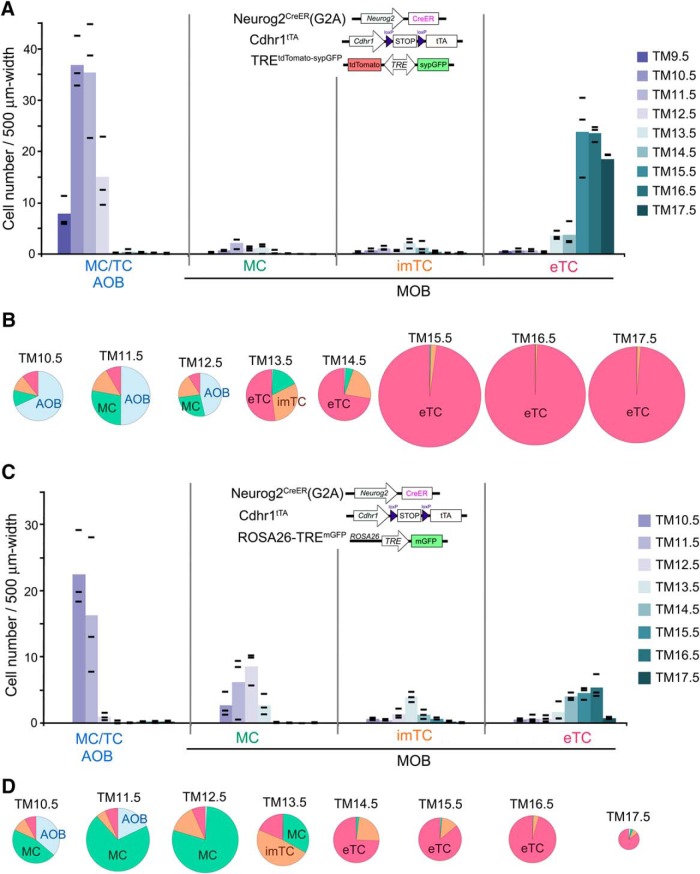
Birthdate tagging of OB projection neurons using OB-specific reporters. The birthdate-tagged projection neuron subtypes were counted in OB sections prepared from P14 Neurog2^CreER^(G2A); Cdhr1^tTA^; TRE^tdTomato-sypGFP^ (***A***, ***B***) and P14–P15 Neurog2^CreER^(G2A); Cdhr1^tTA^; ROSA26-TRE^mGFP^ (***C***, ***D***) mice that had been given TM injection at different embryonic stages. ***A***, ***C***, The color intensity coded columns show the means of tagged cell numbers at individual TM injection stages. Black bars depict the raw values obtained from individual mice (*n* = 3 for each TM stage). The cell numbers are normalized by the layer distance used for cell counting. ***B***, ***D***, Proportion of neuron subtypes birthdate tagged at different TM stages in the whole OB. The proportions were calculated without normalization by the tissue size, and therefore may look slightly different from the quantifications in ***A***, ***C***. The diameter of each pie chart reflects the number of neurons. The exact numbers of neurons counted are shown in Extended Data Figure 3-1.

P14 or P15 OBs were coronally cut into serial 20 μm-sections. A series of every ten sections were immunostained with rabbit anti-β-gal antibody after the antigen retrieval for Tau^mGFP-nLacZ^ reporter (Figs. 1*D*,*G*, 2*A*,*B*) and with rabbit anti-GFP antibody for ROSA26-TRE^mGFP^ reporter (Fig. 3*C*,*D*). For the TRE^tdTomato-sypGFP^ reporter (Fig. 3*A*,*B*), tdTomato-positive cells were counted without antibody staining. MCs were defined as the cells whose cell bodies were positioned in the MC layer (MCL); imTCs in the deep 2/3rd of the EPL; eTCs from the upper 1/3rd of the EPL to the lower 1/3rd of the GLL. The number of the reporter-tagged neuron subtypes within a 500 μm-width of OB layer was manually counted under a fluorescent microscope (Axioplan2. Zeiss) with a 20× objective lens (FLUAR). For each single mouse, 42–74, 37–81, and 37–81 visual fields were counted bilaterally from the entire OBs of Tau^mGFP-nLacZ^ (Fig. 1*D*,*G*), TRE^tdTomato-sypGFP^ (Fig. 3*A*,*B*) and ROSA26-TRE^mGFP^ (Fig. 3*C*,*D*) reporter lines, respectively. The numbers of neuron subtypes were then summed up for each mouse and normalized by the dimension of the examined layers, leading to the value per 500-μm width of layer (Figs. 1*D*, 3*A*,*C*). The proportion of OB neuron subtypes was calculated without normalization by the tissue dimension using the datasets of three mice (Figs. 1*G*, 3*B*,*D*), so that it represents the total cell composition in the whole OB. The exact numbers of neurons counted are shown in Extended Data Figures 1-1, 3-1.

10.1523/ENEURO.0234-19.2019.f3-1 Extended Data Figure 3-1 Number of neurons counted in Fig.3. Download Figure 3-1, TIF file.

For distribution analyses of birthdate-tagged neurons (Fig. 2*A*,*B*), the number of β-gal-positive MCs and eTCs were counted from the rostral, dorsal, lateral, medial, and ventral sectors of MOBs. The rostral counts were from coronal sections positioned 200–600 μm from the rostral end of the OB. The other counts were from the most dorsal, lateral, medial and ventral visual fields placed in each OB coronal section at the level of 1000–2800 μm from the rostral end.

The proportion of birthdate-tagged neurons that expressed neuron subtype markers (Fig. 2*C–F*) was calculated by manually counting the neurons in MOB sections of three P14 mice with a 40× objective lens (Plan-Apochromat) under a fluorescent microscope (Zeiss Axioplan2).

### Fluorescent dextran labeling

Whole brains were dissected in Hanks’ balanced salt solution from P14.5 Neurog2^CreER^(G2A); Cdhr1^tTA^; TRE^tdTomato-sypGFP^ triple heterozygotes that had been given TM at E15.5. A small crystal of tetramethylrhodamine or fluorescein-conjugated fixable dextran (Thermo Fisher catalog #D3308, D3306) was prepared following the described methods (Glover, 1995; Sato et al., 1998) and inserted into the anterior most position of the OT that was accumulated with tdTomato-expressing axons under a fluorescence dissection microscopy (MZFLII, Leica). Until the dextran was transported through the axons, the brains were roll-bottle cultured for 3–4 h at 37°C in Neurobasal medium (Thermo Fisher) containing B27 supplements (Thermo Fisher) in an atmosphere of 95% O_2_/5% CO_2_ using a whole embryo culture apparatus (10-310, Ikemoto Rica Kogyo). The brains were then fixed with 4% PFA/PBS and subjected for histochemical analyses as described above. The dextran injections were performed on the both sides of 12 brains, and a confined labeling of the targeted cell cluster in the most anterolateral OT was successful in nine brains (at least one side). In all these successful cases, a small bundle of dextran-labeled axons coursing through the polymorph layer of the OT was observed as shown in the Figure 12*C*,*D*.

### Virus labeling

AAV2-TRE^TurboFP635^ (3.0 × 10^12^ vg/ml) and VSVG (Lenti)-TRE^GFP^ (4.0 × 10^12^ vg/ml) were prepared as described previously (Kato et al., 2007, 2018). Neurog2^CreER^(G2A); Cdhr1^tTA^ double heterozygotes mice (two to five months of age) that had been given TM at E15.5 were anesthetized with medetomidine hydrochloride (0.3 mg/kg) and sodium pentobarbital (20 mg/kg), and head-fixed in an apparatus (EDMS11-054 SR-5M-S, Narishige) under a dissection microscopy. A small area of the bone above the OB was carefully cut out using a microgrinder (UC210, Urawa) with a diamond point (ED101). A pulled borosilicate glass pipette (3B100-75-10, Sutter Instrument) filled with the virus solution was placed in a holder (HI-7, Narishige) on a manual course manipulator (Narishige) and inserted into the medial or lateral side of the MOB according to the atlas of the mouse brain (Paxinos and Franklin, 2008). More specifically, the pipette was pierced through the midline of each OB positioned between 1/3 and 2/3 distance along the anterior-posterior axis, avoiding blood vessels, and penetrated medially for 75–100 μm or laterally for 125–150 μm at ∼60° oblique angle to the surface plane. The virus solution (1 μl) was injected slowly over 10 min using an oil syringe injector (IM-6, Narishige). The window over the OB was then covered with the removed bone and sealed with Vetbond Tissue Adhesive (3M Science #1469). At three to four weeks after the virus injection, the brains were dissected out for histochemical examination. The double virus injections were performed unilaterally to the OB of 13 mice, among which four were successful for focal labeling of both of the viruses and exhibited qualitatively same results as shown in Figure 13.

### Statistical analysis

The exact numbers of animals used in individual experiments were described in the figure legends or methods. Numerical values were calculated using Microsoft Excel (Mac 2011, Microsoft). No data were excluded for the quantification. Due to the limitation in the number of mice with the appropriate combination of the recombinant genes, sample sizes of this study were inevitably small, and therefore, all the individual data points were represented in graphs for accuracy (Figs. 1*C*,*D*, 2A,*B*, 3*A*,*C*). Because this study does not need any dichotomous judgment, null hypothesis significance testing involving *p* value was not performed. When the sample size is sufficiently large (Fig. 1*C*), bootstrap confidence was inferred by the estimation statistics (https://www.estimationstats.com/; Ho et al., 2019).

### Availability of data and materials

All data and materials generated in this study are available from the corresponding author on reasonable request. The mouse lines generated in this study are deposited at RIKEN for BDR (http://www2.clst.riken.jp/arg/TG%20mutant%20mice%20list.html) and provided under materials transfer agreements.

## Results

### Generation of birthdate tag driver mouse

The birthdate tagging takes advantage of neuronal differentiation genes, which are expressed within a short time window immediately after neurons exit their final cell cycle (Fig. 1*A*). For example, the expression of a basic helix-loop-helix transcription factor, Neurog2, is sharply up-regulated in neuronally committed cells, sustained during the neuronal differentiation phase, and then down-regulated in the maturing phase (Shimojo et al., 2008; Ochiai et al., 2009; Telley et al., 2016). Using enhancers of such neuronal differentiation genes, the TM-inducible Cre recombinase, or CreER gene (Feil et al., 1997) has been expressed transiently in newly born neurons in several genetically modified mice (Fig. 1*A*; Zirlinger et al., 2002; Battiste et al., 2007; Kim et al., 2011; Winpenny et al., 2011). Although these mice have been primarily used for cell lineage analyses related to the manipulated genes (Zirlinger et al., 2002; Ma and Wang, 2006; Battiste et al., 2007; Kim et al., 2011; Florio et al., 2012), they are theoretically applicable for neuronal birthdating (Sudarov et al., 2011; Toma et al., 2014), because a single administration of TM at a certain developmental stage is expected to induce recombination of loxP sequences only in the CreER-expressing neurons that are in the postmitotic differentiation phase (Fig. 1*A*).

Because the lineage of OB projection neurons expresses Neurog2 (Schuurmans and Guillemot, 2002), we first tested an existing mouse line in which the endogenous Neurog2 gene is replaced by the CreER gene (Zirlinger et al., 2002). However, even with a sufficient dose of TM, the Cre-loxP recombination efficiency was too low for birthdating analyses of OB neurons. Thus, we decided to generate a more efficient mouse line aimed at neuronal birthdating by a brute-force transgenic approach using a potential enhancer of Neurog2. We generated many transgenic mice and successfully isolated a birthdate tag driver line, Neurog2^CreER^(G2A) (Fig. 1*B*; Table 1), that robustly induces loxP recombination in a TM-dependent manner.

### Birthdate tagging of OB projection neurons

We first tested whether the Neurog2^CreER^(G2A) line would work for birthdate labeling of OB neurons using a global neuronal Cre reporter, Tau^mGFP-nLacZ^ (Hippenmeyer et al., 2005), which expresses dual nucleus- and membrane-anchored reporters in widespread neurons on loxP recombination (Fig. 1*B*; Table 1). When the Neurog2^CreER^(G2A) mouse was crossed with the Tau^mGFP-nLacZ^ reporter, a TM injection during gestation stages was successful in labeling different types of OB neurons depending on the injection stage (Fig. 1*D–G*). More specifically, projection neurons of the AOB were selectively labeled from the very earliest TM injection at E10.5–E11.5 (hereafter called TM10.5-11.5; Fig. 1*D*). The labeling of MCs in the MOB peaked at TM12.5. Neurons labeled by subsequent injections gradually shifted from deeper to more superficial surfaces of the EPL (Fig. 1*E*). Judging from the location of their cell bodies (Fig. 1*E*) and dendrites (Fig. 1*F*; Mori et al., 1983; Orona et al., 1984), imTCs and eTCs were most effectively labeled at TM13.5 and TM15.5, respectively (Fig. 1*D*). Figure 1*G* shows the estimated proportion of OB neuron subtypes tagged at individual TM stages in the whole OB.

To characterize the cell-cycle state of OB neurons that underwent TM-induced recombination, we conducted a double injection of TM at the fixed E12.5 stage and a thymidine-analog EdU at a certain time point either before or after the TM injection (Fig. 1*C*). The emergence of EdU and reporter double-positive neurons indicated that OB neurons were most susceptible to the TM-induced recombination 6–12 h after final DNA synthesis (Fig. 1*C*). Taking this time lag into account, the time course of the TM-induced OB labeling (Fig. 1*D*,*G*) matches well with the results of previous neuronal birthdating studies using ^3^H-thymidine in OB neurons (Hinds, 1968; Bayer, 1983; Grafe, 1983).

We next examined the spatial distribution of the birthdate-tagged neurons along the tangential plane of different MOB sectors (Fig. 2*A*,*B*). Unlike a previous report describing enrichment of late-born MCs in the ventral MOB (Imamura et al., 2011), the reporter-tagged MCs (Fig. 2*A*) and eTCs (Fig. 2*B*) were distributed in a more or less random manner across all MOB sectors regardless of the time of TM injection.

Lastly, neuron marker analyses confirmed that the TM-tagged neurons indeed belong to the MOB projection neuron subtypes. Most TM12.5-, TM13.5-, and TM15.5-tagged neurons expressed the excitatory projection neuron marker TBR2 (Mizuguchi et al., 2012; Stahl et al., 2016; TM12.5: 99.2 ± 0.4%, TM13.5: 99.6 ± 0.3%, TM15.5: 100.0 ± 0.0%, mean ± SEM; Fig. 2*C–E*). The majority were also positive for cholecystokinin, a widely used but less clear-cut immunohistochemical marker for both MCs and TCs (Seroogy et al., 1985; Cheetham et al., 2015; TM12.5: 64.8 ± 5.7%, TM13.5: 71.6 ± 6.5%, TM15.5: 66.5 ± 5.9%; Fig. 2*C–E*). None of these TM-tagged neurons expressed interneuron markers (Kosaka et al., 1998; Allen et al., 2007; Fig. 2*C–E*). The TM17.5-tagged neurons (Fig. 2*F*) were also positive for TBR2 (100.0 ± 0.0%) but less positive for cholecystokinin (16.1 ± 2.3%); many of the reporter-tagged cells were distributed over a surface area beyond the cholecystokinin-positive cell band at the boundary between the EPL and GLL (Fig. 2*F*). However, because none expressed the OB interneuron markers (Fig. 2*F*), it seems generally acceptable to classify these cells as a subset of eTCs based on their cell body positions (Macrides and Schneider, 1982; Hayar et al., 2004b; Antal et al., 2006). Taken together, these results indicate that birthdate tagging indeed subclassifies MOB projection neurons.

### Mouse lines to visualize axon trajectories of birthdate-tagged OB neurons

Our next focus was axon trajectories of the birthdate-tagged OB projection neurons completed in adult mice. Because labeling of neurons outside the OB interferes with the observation of labeled axons projected out of the OB, we introduced an OB-specific transgene Cdhr1^tTA^, by which the tetracycline-controlled transactivator tTA is expressed in an OB neuron-specific manner on excision of the loxP-flanked sequence (Fig. 3; Table 1). The expression of tTA was then reported with amplification using its responsive TRE reporters, TRE^tdTomato-sypGFP^ (Li et al., 2010; Fig. 3*A*) or ROSA26-TRE^mGFP^ (Fig. 3*C*). Thus, three genes including the birthdate tag driver Neurog2^CreER^(G2A) were combined (Fig. 3*A*,*C*) to specifically visualize birthdate-tagged OB axons.

This genetic design contained two caveats. First, the Cdhr1 enhancer is not completely OB-specific (Nagai et al., 2005); a small fraction of neurons in olfactory target areas were occasionally labeled intensely with the reporter depending on TM injection stages. Second, the two TRE reporters marginally differ in labeling of OB neurons even in the same combination with the other two genes (Fig. 3). Specifically, using the TRE^tdTomato-sypGFP^ reporter, MCs of the MOB were only faintly labeled and their axons were hardly detected, whereas AOB neurons and eTCs were intensely labeled (Fig. 3*A*,*B*). This may be due to cell-type specific epigenetic silencing of the TRE in transgenes as suggested by previous reports (Zhu et al., 2007; Oyer et al., 2009). The ROSA26-TRE^mGFP^ reporter, in contrast, nicely visualized MCs of the MOB, but counted fewer numbers of AOB neurons and eTCs (Fig. 3*C*,*D*). We assume that these numbers are likely to be underestimated in the quantification, because this membrane-anchored GFP reporter only weakly labels cell bodies while intensely labeling fibers, thereby greatly obscuring cell bodies of AOB neurons and eTCs that are buried in fiber-dense regions. Each reporter thus has its drawback and advantage, but, more importantly, the labeling of OB subtypes was strictly TM-dependent and reproducible in each reporter line. Furthermore, the overall time course of the subtype labeling was consistent with that using the global Tau^mGFP-nLacZ^ reporter (Fig. 1*D*,*G*). Therefore, by using both reporters and carefully comparing results, we conducted trajectory analyses of birthdate-tagged OB neurons.

### Axon trajectories of TM10.5-tagged OB neurons

**Figure 4.**
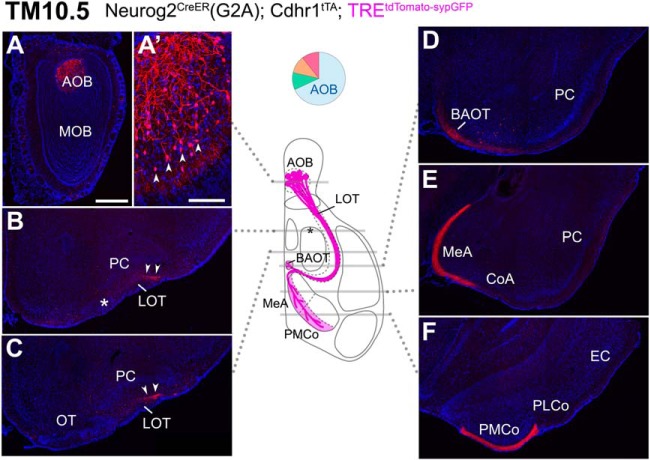
Birthdate tagging at TM10.5. ***A–F***, Coronal brain sections prepared from a P22 Neurog2^CreER^(G2A); Cdhr1^tTA^; TRE^tdTomato-sypGFP^ mouse that was given TM injection at E10.5. Images for tdTomato reporter (red) and DAPI (blue). Medial is to the left and dorsal is to the top. The TM10.5 tagging selectively labels AOB projection neurons (***A***, arrowheads in ***A’***). Their axons run in the deep dorsal side of the LOT (arrowheads in ***B***, ***C***) and project to the AOB-specific targets (***D–F***). Asterisks indicate the position of the anterolateral edge of the OT. Brain illustration in the middle summarizes TM10.5 axon projections and indicates the levels at which individual sections were prepared. The pie chart shows the proportion of neuron subtypes tagged at this TM stage with this reporter. Representative images from five mice. Scale bar = 100 μm (***A’***), 500 μm (***A–F***). BAOT: bed nuclei of the accessory olfactory tract, CoA: cortical amygdala, EC: entorhinal cortex, MeA: medial amygdala, PLCo: posterolateral cortical amygdala, PMCo: posteromedial cortical amygdala.

Using the TRE^tdTomato-sypGFP^ reporter, the birthdate tagging at TM10.5 visualized the specific AOB trajectories (Fig. 4). On this early TM injection, the projection neurons in the AOB were selectively labeled (Fig. 4*A*,*A”*), and their axons projected almost exclusively to the known AOB target areas (von Campenhausen and Mori, 2000; Mohedano-Moriano et al., 2007). More specifically, the TM10.5 axons clearly coursed through the deep dorsal part of the lateral olfactory tract (LOT; Fig. 4*B*,*C*, arrowheads), as reported before (Inaki et al., 2004), and reached all the AOB-specific targets (BAOT, MeA, and PMCo in Fig. 4*D–F*), but no other areas. When the ROSA26-TRE^mGFP^ reporter was instead used for visualization, axons were found to project to both of the AOB and MOB targets, displaying a similar pattern to that of TM11.5 axons with the same reporter that will be described next.

### Axon trajectories of TM11.5-tagged OB neurons

**Figure 5.**
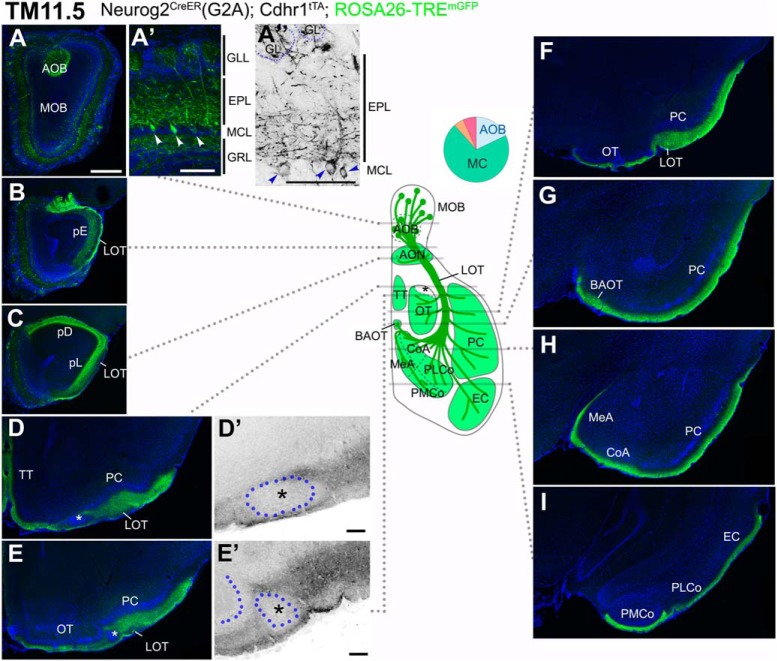
Birthdate tagging at TM11.5. ***A–I***, Coronal brain sections prepared from a P21 Neurog2^CreER^(G2A); Cdhr1^tTA^; ROSA26-TRE^mGFP^ mouse that was given TM at E11.5. Images for mGFP reporter (green) and DAPI (blue). ***A”***, ***D’***, ***E’***, Black and white high contrast image converted from the mGFP image. ***A’***, ***A”***, Higher magnifications of the MOB, of which layer positions are indicated on the right-hand end. Arrowheads (***A’***, ***A”***) indicate cell bodies of birthdate-tagged MCs. Note that the basal dendrites in the lower part of the EPL are labeled with the reporter. The glomerulus (GL) is encircled by a dotted line (***A”***). The TM11.5 axons project diffusely to all the MOB and AOB targets (***B–I***) except the small domain in the anterolateral edge of the OT (asterisks in ***D***, ***D’***, ***E***, ***E’***). ***D’***, ***E’***, Position of a cell cluster is encircled by the blue dotted line and marked with the asterisk. The curved dotted line on the left side of the cell cluster in ***E’*** depicts the lateral hook of the OT cell layer. The labeling of the LOT axons looks less intense possibly because the axons are highly myelinated (Inaki et al., 2004). The brain illustration in the middle summarizes TM11.5 axon projections and indicates the levels at which individual sections were prepared. A pie chart shows the proportion of neuron subtypes tagged at this TM stage with this reporter. Representative images from six mice. Scale bars = 100 μm (***A’***, ***A”***, ***D’***, ***E’***) and 500 μm (***A–I***). BAOT: bed nuclei of the accessory olfactory tract, CoA: cortical amygdala, EC: entorhinal cortex, GRL: granule cell layer, MeA: medial amygdala, pD: pars dorsalis, pL: pars lateralis, PLCo: posterolateral cortical amygdala, PMCo: posteromedial cortical amygdala, TT: tenia tecta.

With TM11.5 tagging, the TRE^tdTomato-sypGFP^ reporter still visualized the AOB-specific projections as those of TM10.5 (Fig. 4). The ROSA26-TRE^mGFP^ reporter instead visualized the widespread mixed projections from both the AOB and MOB (Fig. 5). Specifically, in the OB, AOB neurons and MCs of the MOB were both intensely labeled (Fig. 5*A*). The labeled basal dendrites in the MOB were confined to the deep part of the EPL (Fig. 5*A’*,*A”*), confirming the labeling of MCs (Mori et al., 1983; Orona et al., 1984). Their axons covered all the widespread olfactory areas (Fig. 5*B–I*) including both of the AOB and MOB targets (Schwob and Price, 1984; Hintiryan et al., 2012). There was, however, one exceptional vacant space that excluded these axons in the anterolateral edge of the OT abutting the LOT (Fig. 5*D*,*D’*,*E*,*E’*, asterisks), which we will focus on later.

### Axon trajectories of TM12.5-tagged OB neurons

**Figure 6.**
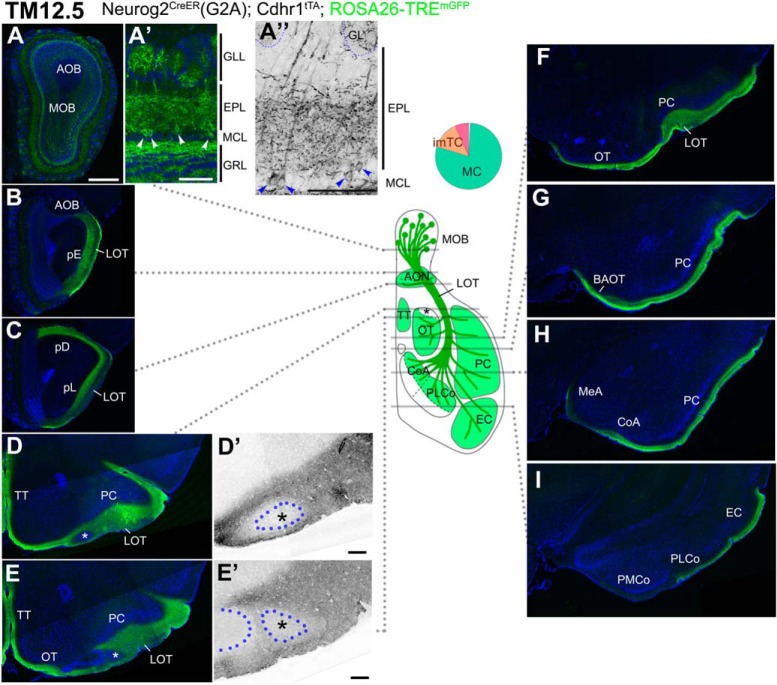
Birthdate tagging at TM12.5. ***A–I***, Coronal brain sections prepared from a P20 Neurog2^CreER^(G2A); Cdhr1^tTA^; ROSA26-TRE^mGFP^ mouse that was given TM at E12.5. Images for mGFP reporter (green) and DAPI (blue). ***A”***, ***D’***, ***E’***, Black and white high contrast image converted from the mGFP image. ***A’***, ***A”***, Higher magnifications of the MOB, of which layer positions are indicated on the right-hand end. Arrowheads (***A’***, ***A”***) indicate cell bodies of birthdate-tagged MCs. Note that the basal dendrites in the lower part of the EPL are labeled. The glomerulus (GL) is encircled by a dotted line (***A”***). The TM11.5 axons project diffusely to all the MOB targets (***B–I***) except the small domain in the anterolateral edge of the OT (asterisks in ***D***, ***D’***, ***E***, ***E’***). ***D’***, ***E’***, Position of a cell cluster is encircled by the blue dotted line and marked with the asterisk. The curved dotted line on the left side to the cell cluster in ***E’*** depicts the lateral hook of the OT cell layer. A brain illustration in the middle summarizes TM12.5 axon projections and indicates the levels at which individual sections were prepared. A pie chart shows the proportion of neuron subtypes tagged at this TM stage with this reporter. Representative images from seven mice. Scale bars = 100 μm (***A’***, ***A”***, ***D’***, ***E’***) and 500 μm (***A–I***). BAOT: bed nuclei of the accessory olfactory tract, CoA: cortical amygdala, EC: entorhinal cortex, GRL: granule cell layer, MeA: medial amygdala, pD: pars dorsalis, pL: pars lateralis, PLCo: posterolateral cortical amygdala, PMCo: posteromedial cortical amygdala, TT: tenia tecta.

The TM12.5 tagging using the ROSA26-TRE^mGFP^ reporter brought a more specific labeling of MCs of the MOB, leaving AOB neurons mostly unlabeled (Fig. 6*A*). The basal dendrites in the MOB were more densely labeled, compared with those at TM11.5, but still confined to the deep part of the EPL (Fig. 6*A’*,*A”*), supporting the selective labeling of MCs.

At this TM injection stage, labeling of AOB targets was extremely faint (Fig. 6*G–I*), displaying a complementary image to that of TM10.5 axons (Fig. 4*D–F*). The TM12.5 axons instead projected to all the known MOB target areas (Fig. 6*B–I*; Schwob and Price, 1984; Walz et al., 2006; Hintiryan et al., 2012). Their wide diffuse projections were likely due to extensive branching of these axons as shown previously (Ghosh et al., 2011; Sosulski et al., 2011; Igarashi et al., 2012), and indeed recapitulated the general image of divergent central olfactory projections. However, as observed for the TM11.5 axons, we found a small oval space that excluded the TM12.5 axons and remained unlabeled, in the anterolateral edge of the OT abutting the LOT (Fig. 6*D*,*D’*,*E*,*E’*, asterisks). The wide remaining part of the OT was uniformly and densely filled with TM12.5 axons (Fig. 6*E*,*F*).

### Axon trajectories of TM13.5-tagged OB neurons

**Figure 7.**
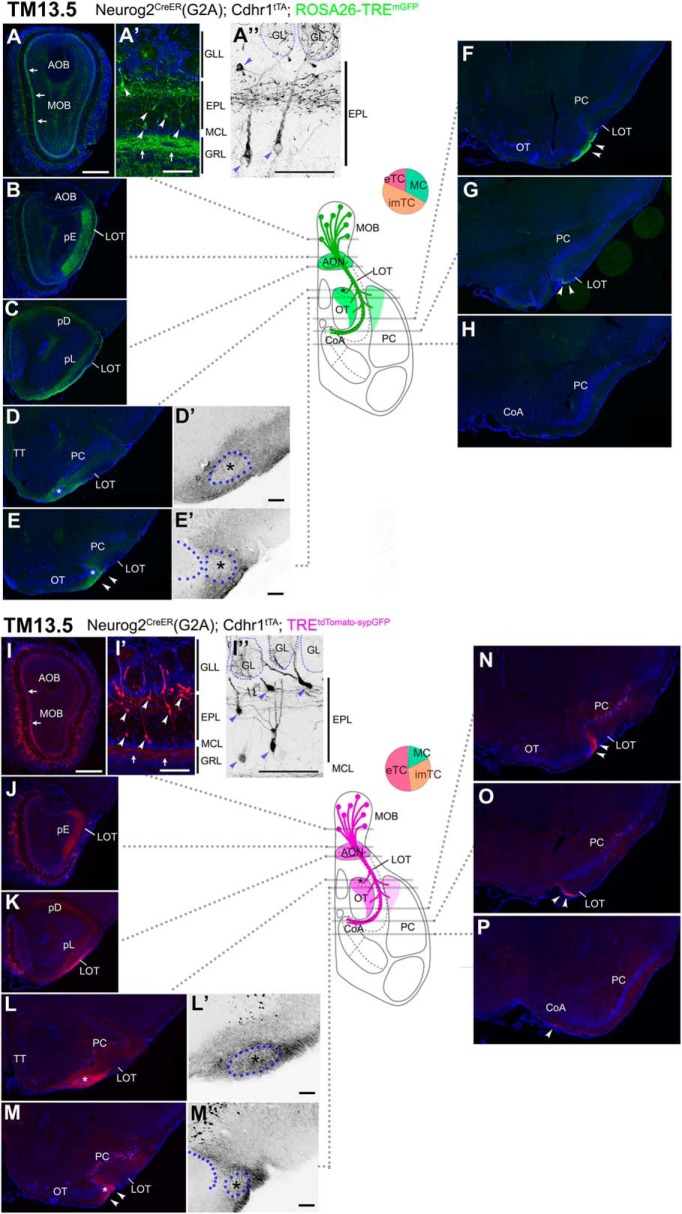
Birthdate tagging at TM13.5. Coronal brain sections prepared from P21 Neurog2^CreER^(G2A); Cdhr1^tTA^; ROSA26-TRE^mGFP^ (***A–H***) and P21 Neurog2^CreER^(G2A); Cdhr1^tTA^; TRE^tdTomato-sypGFP^ (***I–P***) mice that were given TM at E13.5. Images for mGFP reporter (***A–H***) and tdTomato reporter (***I–P***) counterstained with DAPI. ***A”***, ***D’***, ***E’***, ***I”***, ***L’***, ***M’***, Black and white high contrast image converted from the reporter image. ***A’***, ***A”***, ***I’***, ***I”***, High magnifications of the MOB, of which layer positions are indicated on the right-hand end. Arrows (***A***, ***A’***, ***I***, ***I’)*** indicate the internal plexiform layer that contains intrabulbar association fibers. Arrowheads (***A’***, ***A”***, ***I’***, ***I”***) indicate cell bodies of birthdate-tagged imTCs and eTCs. Note that the basal dendrites in the upper part of the EPL are labeled. The glomerulus (GL) is encircled by a dotted line (***A”***, ***I”***). The TM13.5 axons project to only anterior part of the olfactory target areas (***B–H***, ***J–P***). Arrowheads (***E***, ***F***, ***M***, ***N***) indicate axons projecting the lateral part of the OT. Arrowheads (***G***, ***O***) indicate labeled axons reaching to the caudal end of the LOT. Asterisks (***D***, ***D’***, ***E***, ***E’***, ***L***, ***L’***, ***M***, ***M’***) indicate the small domain in the anterolateral edge of the OT. ***D’***, ***E’***, ***L’***, ***M’***, Position of a cell cluster is encircled by the blue dotted line and marked with the asterisk. The curved dotted line on the left side of the cell cluster in ***E’***, ***M’*** depicts the lateral hook of the OT cell layer. At this TM injection stage, scattered cells in the PC express reporters (***E***, ***F***, ***L***–***P***). The brain illustration in the middle summarizes TM13.5 axon projections and indicates the levels at which individual sections were prepared. The pie chart shows the proportion of neuron subtypes tagged at this TM stage in each reporter line. Representative images from eight (***A–F***) and seven (***G–L***) mice. Scale bars = 100 μm (***A’***, ***A”***, ***D’***, ***E’***, ***I’***, ***I”***, ***L’***, ***M’***) and 500 μm (***A–P***). CoA: cortical amygdala, GRL: granule cell layer, pD: pars dorsalis, pL: pars lateralis, TT: tenia tecta.

A notable characteristic of TM 13.5 tagging is the labeling of imTCs, although their proportion among all labeled neurons was still below 50% using either of the reporters (Fig. 3*B*,*D*). Cell bodies of the labeled imTCs were scattered in varying positions across the EPL (Fig. 7*A–A”*,*I–I”*). The labeled basal dendrites, projected from the whole population tagged at this TM stage, were confined to the upper part of the EPL (Fig. 1*A’*,*A”*,*I’*,*I”*), indicating that imTCs and eTCs are the main sources of the dendrites (Mori et al., 1983; Orona et al., 1984). The internal plexiform layer at the most superficial part of the granule cell layer was also labeled (Fig. 1*A*,*A’*,*I*,*I’*, arrows), suggesting that at least some of the TM13.5-tagged population have intrabulbar association fibers, bifurcated branches of OB axons coursing through this layer to connect to the other side of the OB (Schoenfeld et al., 1985; Belluscio et al., 2002).

The axon trajectories visualized with the two reporters appeared more or less similar (Fig. 7*B–H*,*J–P*). Compared with earlier TM injection stages, the projection area was drastically reduced, and covered only the anterior part of the olfactory target areas, namely all the subdivisions of the AON (Fig. 7*B*,*C*,*J*,*K*), the lateral half of the OT (Fig. 7*D–F*,*L–N*), and the anterior part of the PC (Fig. 7*D*,*E*,*L*,*M*). Within the LOT, the reporter-labeled axons, bundling together, coursed through the most ventral part down to the caudal end (Fig. 7*G*,*O*, arrowheads), at which the axons appeared to simply stall without leaving for any target (Fig. 7*H*,*P*). At this TM injection stage, ectopic neurons outside the OB were frequently labeled in olfactory target areas (Fig. 7*E*,*F* or *L–P*). This undesirable labeling interfered with the detailed mapping of OB axons, but the small space in the anterolateral edge of the OT appeared to be weakly accessible to the TM13.5 axons (Fig. 7*D*,*D’*,*E*,*E’*,*L*,*L’*,*M*,*M’*, asterisks). At this point, the two reporters seemed to make a slight difference: in the ROSA26-TRE^mGFP^ reporter, the axons were dense in the surrounding area (Fig. 7*D*,*D’*,*E*,*E’*), whereas in the TRE^tdTomato-sypGFP^ reporter, the axons more densely projected to the core space in the anterolateral edge of the OT (Fig. 7*L*,*L’*,*M*,*M’*).

### Axon trajectories of TM15.5-tagged OB neurons

**Figure 8.**
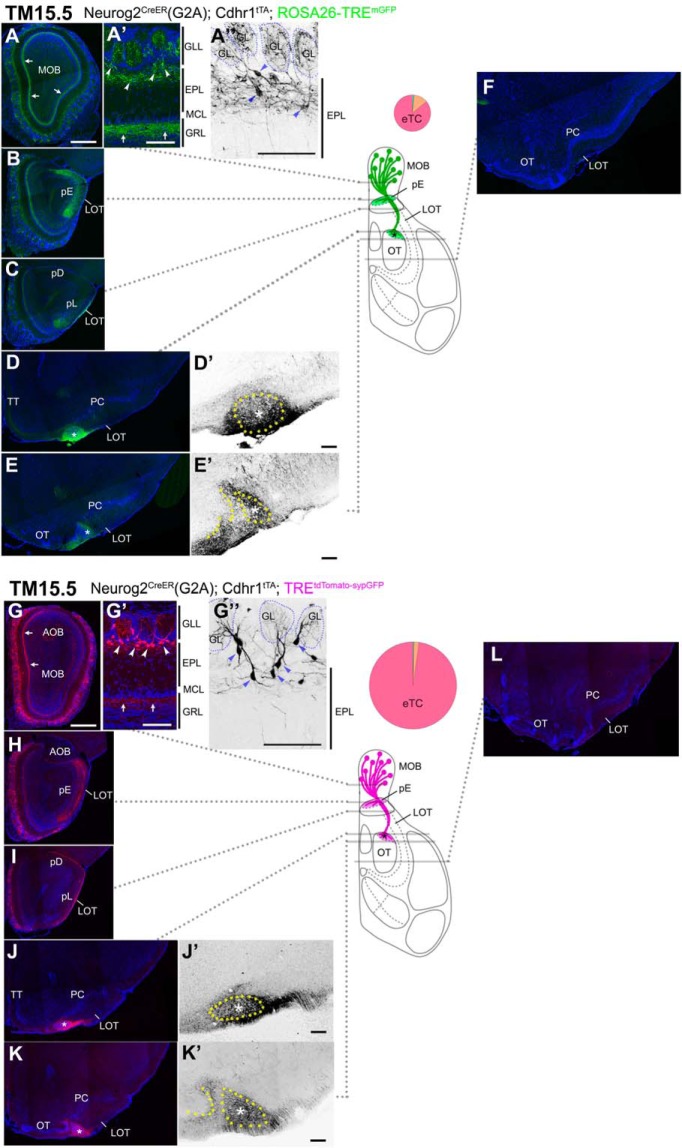
Birthdate tagging at TM15.5. Coronal brain sections prepared from P22 Neurog2^CreER^(G2A); Cdhr1^tTA^; ROSA26-TRE^mGFP^ (***A–F***) and P21 Neurog2^CreER^(G2A); Cdhr1^tTA^; TRE^tdTomato-sypGFP^ (***G–L***) mice that were given TM at E15.5. Images for mGFP reporter (***A–F***) and tdTomato reporter (***G–L***) counterstained with DAPI. ***A”***, ***D’***, ***E’***, ***G”***, ***J’***, ***K’***, Black and white high contrast image converted from the reporter image. ***A’***, ***A”***, ***G’***, ***G”***, High magnifications of the MOB, of which layer positions are indicated on the right-hand end. Arrows (***A***, ***A’***, ***G***, ***G’***) indicate the internal plexiform layer that contains intrabulbar association fibers. Arrowheads (***A’***, ***A”***, ***G’***, ***G”***) indicate cell bodies of birthdate-tagged eTCs, whose basal dendrites horizontally extend in the upper part of the EPL. The glomerulus (GL) is encircled by a dotted line (***A”***, ***G”***). The TM15.5 axons only project to the pE (***B***, ***H***) and the small domain in the anterolateral edge of the OT (asterisks in ***D***, ***D’***, ***E***, ***E’***, ***J***, ***J’***, ***K***, ***K’***). This domain contains a cell cluster, which is encircled by the yellow dotted line and marked with the asterisk (***D’***, ***E’***, ***J’***, ***K’***). The curved dotted line on the left of the cell cluster in ***E’***, ***K’*** depicts the lateral hook of the OT cell layer. The brain illustration summarizes TM15.5 axon projections and indicates the levels at which individual sections were prepared. The pie chart shows the proportion of neuron subtypes tagged at this TM stage in each reporter line. Representative images from five (***A–F***) and nine (***G–L***) mice. Scale bars = 100 μm (***A’***, ***A”***, ***D’***, ***E’***, ***G’***, ***G”***, ***J’***, ***K’***) and 500 μm (***A–L***). GRL: granule cell layer, pD: pars dorsalis, pL: pars lateralis, TT: tenia tecta.

On TM15.5 tagging, eTCs at the boundary between the EPL and GLL were prominently labeled in both reporters (Fig. 8*A*,*A’*,*G*,*G’*). Their cell bodies clung along their basal dendrites that projected horizontally in the upper part of the EPL (Fig. 8*A’*,*A”*,*G’*,*G”*). Although Individual neurons exhibited substantially different morphologies, e.g., horizontally-oriented cells with only one horizontal basal dendrite or small pyramidal ones with multiple basal dendrites (Fig. 8*A”*,*G”*), all these characteristics appeared to belong to a subclass of eTCs (Macrides and Schneider, 1982; Orona et al., 1984; Hayar et al., 2004a; Antal et al., 2006; Takahashi et al., 2016). The internal plexiform layer that contains intrabulbar association fibers was intensely labeled at this TM injection stage (Fig, 8*A*,*A’*,*G*,*G”*, arrows).

**Figure 9.**
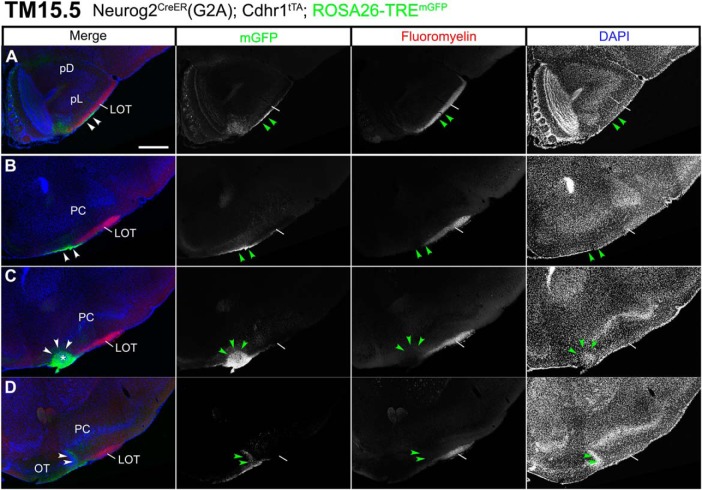
Segregated projections of TM15.5 axons to the target cell cluster in the anterolateral OT. ***A–D***, Coronal brain sections prepared from a P22 Neurog2^CreER^(G2A); Cdhr1^tTA^; ROSA26-TRE^mGFP^ mouse that was given TM at E15.5. Images for mGFP reporter (green), FluoroMyelin (red) and DAPI (blue). FluoroMyelin marks myelinated OB axons running through the LOT. The TM15.5 axons (arrowheads) ventrally segregate from other OB axons in the LOT (***A***, ***B***) and terminate at a small cell cluster in the anterolateral edge of the OT (arrowheads and asterisk in ***C***). In a posterior section (***D***), the cell cluster fuses with the lateral hook of the OT dense cell layer (arrowheads). Representative images from five mice. Scale bars = 500 μm (***A–D***). pD: pars dorsalis, pL: pars lateralis.

The TM15.5 OB axons showed remarkable convergence onto only two small domains within the wide olfactory target areas, regardless of which reporter was used (Fig. 8*B–F*,*H–L*). One is that small space in the anterolateral edge of the OT abutting the LOT (Fig. 8*D*,*D’*,*E*,*E’*,*J*,*J’*,*K*,*K’*), which excluded TM11.5 and TM12.5 axons. At closer inspection, the 15.5 axons arose from the entire MOB (Fig. 8*A*,*G*), ran for a short distance along the surface of the LOT (Fig. 9*A*), shifted ventrally from the other LOT axons (Fig. 9*B*), and finally reached this space (Fig. 9*C*). Here, the dense axon terminals embraced a small, discrete cluster of cells (Figs. 8*D’*,*J’*, dotted circles, 9*C*, arrowheads). This cell cluster was evident in DAPI staining and identifiable based on its unique location abutting the LOT, but has not been described so far. In anterior coronal sections, this target of TM15 axons appeared as one or two isolated cell clusters medially adjacent to the LOT underneath the arched cell layer of the PC (Figs. 8*D*,*J*, 9*C*). Posteriorly, it was positioned just laterally next to the lateral end of the OT cell layer that bent dorsally like a hook, forming a sidewall of the LOT (Fig. 8*E*,*E’*,*K*,*K’*), and eventually fused with this hook of the OT cell layer at a more posterior level (Fig. 9*D*, arrowheads). The wide remaining part of the OT did not seem to receive the TM15.5 axon projections (Fig. 8*E*,*F*,*K*,*L*).

In addition to this new target cell cluster, the TM15.5 axons projected onto a second target, the pE in the AON (Fig. 8*B*,*H*). The AON is a major olfactory target consisting of five subdivisions of unknown function. The four subdivisions other than the pE did not seem to be innervated by the TM15.5 axons (Fig. 8*C*,*I*), whereas all five subdivisions seemed to be innervated by TM11.5-13.5 axons (Figs. 5–7); it is however not possible to precisely distinguish the axons that terminate on the pE from those that simply penetrate the pE to terminate on the juxtaposed pars lateralis (Scott et al., 1985).

### Axon trajectories of TM17.5-tagged OB neurons

**Figure 10.**
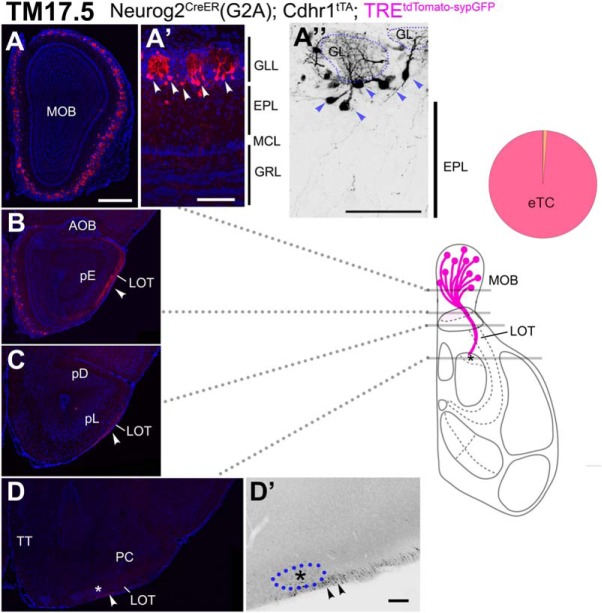
Birthdate tagging at TM17.5. ***A–D***, Coronal brain sections prepared from a P21 Neurog2^CreER^(G2A); Cdhr1^tTA^; TRE^tdTomato-sypGFP^ (***A–D***) mouse that was given TM at E17.5. Images for tdTomato reporter and DAPI. ***D’***, Black and white high contrast image converted from the tdTomato reporter image. ***A’***, ***A”***, High magnifications of the MOB, of which layer positions are indicated on the right-hand end. Arrowheads (***A’***, ***A”***) indicate cell bodies of birthdate-tagged eTCs that barely have basal dendrites. The glomerulus (GL) is encircled by a dotted line (***A”***). A small number of TM17.5 axons project in the ventral surface of the LOT (arrowheads in ***B–D***). Asterisks (***D***, ***D’***) show the anterolateral edge of the OT. The cell cluster encircled by the blue dotted line (***D’***) is not penetrated by TM17.5 axons (arrowheads). The brain illustration summarizes TM17.5 axon projections and indicates the levels at which individual sections were prepared. The pie chart shows the proportion of neuron subtypes tagged at this TM stage in this reporter line. Representative images from seven mice. Scale bars = 100 μm (***A’***, ***A”***, ***D’***) and 500 μm (***A–D***). GRL: granule cell layer, pD: pars dorsalis, pL: pars lateralis, TT: tenia tecta.

The TM17.5 tagging labeled eTCs that were more superficially positioned than those labeled at TM15.5 (Fig. 10*A*,*A”*). Most of these neurons did not have basal dendrites (Fig. 10*A”*) and resembled to another subset of eTCs described previously (Hayar et al., 2004b; Antal et al., 2006; Takahashi et al., 2016). The labeling of intrabulbar association fibers was not evident at this TM injection stage (Fig. 10*A*,*A’*). The TM17.5 axons projected out of the OB only in the anterior part of the LOT (Fig. 10*B*,*C*). These axons passed close to the pE (Fig. 10*B*), and a fraction of them even approached the cell cluster at the anterolateral edge of the OT (Fig. 10*D*,*D’*), but did not take off the tract and penetrate into any of the target areas.

### Characterization of the target cell cluster of TM15.5 axons

**Figure 11.**
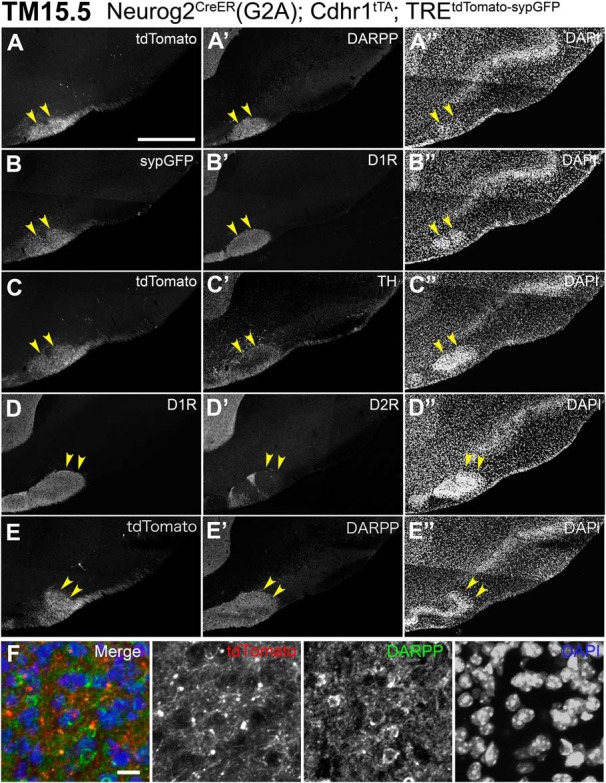
Histochemical characteristics of the target cell cluster of TM15.5 axons. ***A–E***, Five serial coronal sections (20 μm thick), from anterior (***A***) to posterior (***E***), prepared from a P21 Neurog2^CreER^(G2A); Cdhr1^tTA^; TRE^tdTomato-sypGFP^ mouse that was given TM at E15.5. Arrowheads show the cell cluster targeted by TM15.5 axons. The TM15 axons expressing tdTomato (***A***, ***C***, ***E***) and synaptophysin-GFP (***C***) reporters densely project to the target cell cluster stained with DAPI (***A”–E”***). The target cell cluster expresses DARPP (***A’***, ***E’***), dopamine receptors D1R (***B’***, ***D***), but not D2R (***D’***). Dopaminergic axons expressing tyrosine hydroxylase (***C’***) project over this cell cluster. Representative images from three mice. ***F,*** Enlarged image of neurons in the cell cluster, which express DARPP (green) and are projected by the tdTomato-expressing TM15.5 axons (red). Scale bars = 500 μm (***A–E***) and 10 μm (***F***).

We next characterized the newly identified target of TM15.5 axons in the anterolateral OT. Although typical olfactory targets consist of excitatory principal neurons, the OT is a part of the basal ganglia populated with GABAergic spiny neurons that receive dopamine signals (Millhouse and Heimer, 1984; Murata et al., 2015). The cell cluster targeted by TM15.5 axons strongly expressed dopamine-regulated neuronal phosphoprotein-32 (DARPP), a dopamine-responsive neuron marker (Fig. 11*A*,*E*), confirming that it is indeed a component of the OT area. This cell cluster also strongly expressed dopamine receptor D1, but lacked D2 receptor expression (Fig. 11*B*,*D*). Dopaminergic axons presumably from the ventral tegmental area (Fallon et al., 1978; Haberly and Price, 1978a; Fallon, 1983; Mooney et al., 1987) projected over this cell cluster as well as the other remaining parts of the OT (Fig. 11*C*). All these observations suggest that this target cell cluster receives dual inputs, one from the dopaminergic system and the other from the glutamatergic olfactory system through a subset of eTCs, and integrates the information in a similar manner to the striatal direct pathway.

Anatomically, the OT exhibits a chimeric architecture consisting of the cell-layered cortical part, the CAP compartments, and the Calleja islands (Fallon et al., 1978; Meyer and Wahle, 1986; Murata et al., 2015). The D2 receptor-negative characteristic of the target cluster of TM15.5 indicates that it belongs to the CAP compartments (Murata et al., 2015). CAP compartments are defined as about a dozen of periodic grooves toward which the OT cell layer protrudes like ripples approaching and touching the brain surface (Hosoya and Hirata, 1974; Meyer and Wahle, 1986; Murata et al., 2015). Accordingly, each CAP is observed as a brain surface patch that often accompanies a single Calleja island on its bottom. Our observations, taken together, indicate that the target cell cluster of TM15.5 axons represents the most anterolateral isolation of the CAP compartments (aiCAP). Thus, we hereafter refer to this target as aiCAP, although it remains unclear how it was classified in previous studies, due to its atypical isolated configuration. Indeed, at higher magnification, neurons in the aiCAP were simply packed (Fig. 11*F*) and were not associated with a Calleja island or in direct contact with the brain surface.

**Figure 12.**
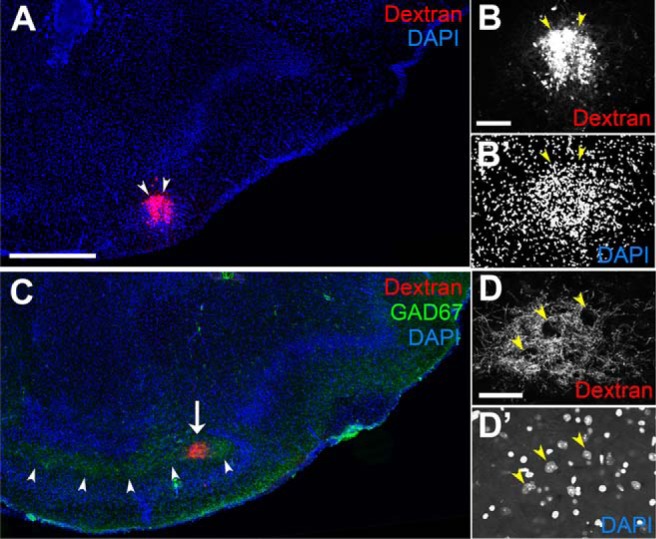
Afferent projections from aiCAP, the target cell cluster of TM15.5 axons. ***A***, Rhodamine-dextran focally injected into the aiCAP (arrowheads) of a P14.5 Neurog2^CreER^(G2A); Cdhr1^tTA^; TRE^tdTomato-sypGFP^ mouse that was given TM at E15.5. ***B***, ***B’***, Enlarged image of the aiCAP (arrowheads) labeled with dextran and DAPI. ***C,*** In a posterior section, the dextran-labeled axons make a small fascicle (arrow) coursing posteriorly through the GAD67-expressing plexus (arrowheads) in the OT polymorph layer. ***D***, ***D’***, High magnification of the dextran-labeled fibers (***D***), which surround neurons (yellow arrowheads), sparsely embedded in the fascicle (***D’***). Representative images from nine mice in which the aiCAP was successfully labeled with dextran. Scale bars = 500 μm (***A***, ***C***), 100 μm (***B***, ***B’***), and 50 μm (***D***, ***D’***).

To assess where the aiCAP projects, we focally injected fluorescent dextran, an anterograde axon tracer, into this cell cluster (Fig. 12*A*,*B*), visually guided by the dense accumulation of TM15.5-tagged axons. The afferent axons of the aiCAP projected posteriorly, creating a small fascicle within the GAD67-expressing plexus in the polymorph layer of the OT (Fig. 12*C*), the area suggested to be analogous to the globus pallidus in the basal ganglia (Young et al., 1984; Heimer et al., 1987). While the dextran-labeled axons projected posteriorly for 300–600 μm in distance, they densely surrounded neurons that were sparsely embedded in the fascicle (Fig. 12*D*). These local neurons may be the target of afferent axons of the aiCAP.

### Target map representations by TM15.5 axons

**Figure 13.**
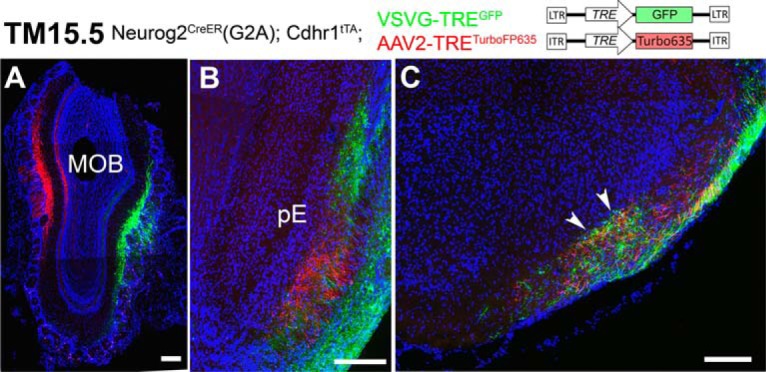
Topographic and non-topographic maps formed by TM15.5 axons. ***A–C***, Coronal sections prepared from a Neurog2^CreER^(G2A); Cdhr1^tTA^ mouse that was given TM15.5 and then injected with AAV2-TRE^TurboFP635^ and VSVG-TRE^GFP^ viruses in the MOB. Medial is to the left and dorsal is to the top. The eTCs on the medial and lateral sides of the MOB are specifically labeled with red and green reporter proteins, respectively (***A***). The axons make topographic projections to the pE (***B***) but not the aiCAP (arrowheads in ***C***). Representative images from four mice with successful focal labeling. Scale bar = 200 μm.

The pE of the AON is the only target in which the odor map is topographically represented (Schoenfeld and Macrides, 1984; Yan et al., 2008). We therefore tested whether the TM15.5 axons were responsible for this topographic projection. In mice that had been birthdate tagged at TM15.5, two reporter viruses, each encoding a different TRE-driven fluorescent protein (Fig. 13), were focally injected into the medial and lateral parts of the MOB, respectively. This procedure brightly and specifically labeled eTCs positioned on the medial and the lateral sides in different colors (Fig. 13*A*). Their axons showed a clear topographic segregation in the pE (Fig. 13*B*), underlining the contribution of TM15.5 axons to the topographic connection from the MOB to the pE. Such a topography was not observed in the aiCAP, within which axon terminals of TM15.5 axons from different MOB regions intermingled (Fig. 13*C*). Therefore, this target cell cluster in the anterolateral OT appeared to contain a whole representation of the MOB map.

### Development of axon projections to the aiCAP

**Figure 14.**
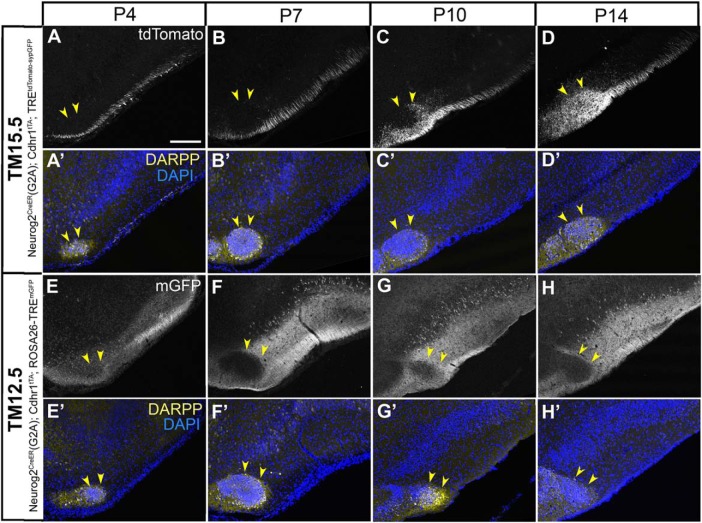
Development of axon projections to the aiCAP. *A-D,* Coronal sections prepared from P4 (***A***), P7 (***B***), P10 (***C***), and P14 (***D***) Neurog2^CreER^(G2A); Cdhr1^tTA^; TRE^tdTomato-sypGFP^ mice that were given TM15.5. ***E-H,*** Coronal sections prepared from P4 (***E***), P7 (***F***), P10 (***G***), and P14 (***H***) Neurog2^CreER^(G2A); Cdhr1^tTA^; ROSA26-TRE^mGFP^ mice that were given TM12.5. Medial is to the left and dorsal is to the top in all panels. Images for tdTomato (***A–D***) and mGFP (***E–H***) reporters. Lower panels (***A’–H’***) show counterstaining images for DAPI and DARPP, which marks the aiCAP (yellow arrowheads) by P4. Scale bar = 200 μm.

Lastly, we examined the development of axon projections to the aiCAP. By P4, this target of TM15.5 axon already begun to express DARPP and was identifiable (Fig. 14*A*). However, the 15.5 axons only penetrated this target from P10 through P14 (Fig. 14*B–D*). The TM12.5 axons, in contrast, began their projections earlier, penetrating the wide olfactory target areas by P4 (Fig. 14*E*). Interestingly, however, even at initial projection stages, these axons were clearly excluded from the aiCAP (Fig. 14*E–H*), which appeared to be saved for later innervation by the TM15.5 axons.

## Discussion

The present study investigated axon trajectories of OB neurons born at different times using a newly developed birthdate tag method. Through comparisons of axon projections from differently mixed populations of OB projection neuron subtypes, a new circuit structure emerged from apparently random central olfactory networks; a subset of eTCs represented by the TM15.5 population form a segregated projection only to two small targets, the pE in the AON and the aiCAP in the OT. The pE appeared to be a convergent target of different projection neuron subtypes, whereas the aiCAP appeared to be a selective, specific target of the eTC subset and to contain a complete representation of the MOB odor map. Although it remains unclear how accessible this target is to other subtypes, the majority of MCs represented by TM11.5 and TM12.5 populations simply avoided projecting to the aiCAP, while the other wide olfactory target areas were closely governed by these neurons. These results indicate that the main olfactory system contains at least two segregated projection pathways branching at the glomeruli.

### Potential information features represented by the eTC pathway

The branched parallel pathway involving multiple representations of the sensory map is a general organizing principle of sensory systems. Owing to this organization, sensory systems can extract unique features of information from a common input (Young, 1998; Nassi and Callaway, 2009). Since the main olfactory system follows this principle, it can also extract qualitatively different information from the common odor map represented in the MOB.

What kind of features can be represented in the eTC channel? The underdeveloped basal dendrites of eTCs have suggested that they receive weaker lateral inhibitions (Macrides and Schneider, 1982; Orona et al., 1984; Antal et al., 2006; Griff et al., 2008a; Economo et al., 2016). Electrophysiological analyses show that eTCs are activated at lower odor concentrations and an earlier time window than MCs (Schneider and Scott, 1983; Ezeh et al., 1993; Fukunaga et al., 2012; Igarashi et al., 2012). Thus, following odor stimulation, eTCs may play a more active role in early processing of odor information, employing their specific targets. Their functions can be significantly different from those of MCs that are activated later after odor stimulation and convey signals to the other wide target areas that can integrate top-down signals, reflecting various behavioral scenes (Shiotani et al., 2018).

Potential functions of the eTC channel can also be inferred from their specific targets. The neurochemical characteristics of the aiCAP suggest that this pathway belongs to the dopamine reward system (Fallon et al., 1978; Haberly and Price, 1978b; Young et al., 1984; Mooney et al., 1987; Wesson and Wilson, 2011; Gadziola et al., 2015). Considering that the connections develop after the juvenile stage (Fig. 14), the related behavioral output may be based on motivation-related olfactory learning, rather than innate functions, as suggested before (Murata et al., 2015). The pE exclusively projects to the MOB on the contralateral side (Haberly and Price, 1978a; Schoenfeld and Macrides, 1984). Through its topographic connections from the ipsilateral MOB, this subnucleus has been suggested to mediate interactions between the two bilateral glomeruli that receive the same odorant receptor input (Yan et al., 2008; Kikuta et al., 2010).

Fortunately, these entire sets of distinct possibilities are now directly testable with the help of the birthdate tag method. In other words, this study provides the basis for future studies at the functional level that would confirm the anatomic observations reported in this study.

### Heterogeneous properties of eTC subtypes

Some previous studies have characterized eTCs as interneurons that do not project out of the OB (Macrides et al., 1985; Schoenfeld et al., 1985; Liu and Shipley, 1994; Nagayama et al., 2014). Based on the present results, we can assume retrospectively that it was extremely difficult to incidentally hit their small targets with a retrograde axon tracer or antidromic electrode stimulation, and that the afferent projections of eTCs may have simply remained undetected in some previous experiments (Haberly and Price, 1977; Skeen and Hall, 1977; Schneider and Scott, 1983).

The present study, at the same time, shows that eTCs are a subdividable population. One subset tagged at TM15.5 contained the projection neurons that send extremely confined afferents to the pE and aiCAP. The E15.5 population had basal dendrites elongating in the upper part of the EPL and dense intrabulbar association fibers (Fig. 8), and more than half were positive for cholecystokinin (Fig. 2*E*). Another subset tagged at TM17.5 seemed to consist of mostly interneurons, although some had axons projecting into the LOT without any ultimate targets. The TM17.5 population barely had basal dendrites or intrabulbar association fibers (Fig. 10), and the majority were cholecystokinin-negative (Fig. 2*F*). Interestingly, these characteristics of eTCs subsets based on their birthdates agree well with those of eTC subgroups classified by other criteria (Macrides and Schneider, 1982; Schoenfeld et al., 1985; Liu and Shipley, 1994; Hayar et al., 2004b; Antal et al., 2006; Takahashi et al., 2016).

### Uncertainties related to imTCs

Projections of imTCs have remained enigmatic in this study, since we are so far unable to separate them from MCs or eTCs using the birthdate tag approach. The imTCs have been considered representative of TCs, and, accordingly, much of our knowledge about TCs is actually derived from imTCs (Mori et al., 1983; Scott et al., 1985; Ezeh et al., 1993; Nagayama et al., 2004; Griff et al., 2008b; Ghosh et al., 2011). A previous study has visualized complete axon trajectories of single individual imTCs associated with the electrically identified glomeruli (Igarashi et al., 2012). Although only a limited number of cells were analyzed, these imTCs show a characteristic distribution of bushy terminals in the pE and pars posterior of the AON as well as clustered terminals along the border between the OT and the medial edge of the anterior PC. Indeed, these terminal domains resemble those of the TM13.5 population that substantially included imTCs (Fig. 7).

Traditionally imTCs have been considered abundant members in MOB projection neurons (Haberly and Price, 1977; Nagayama et al., 2014), but in the present study, imTCs represented only a minor fraction of the birthdate-tagged OB neurons. Before discussing this seeming discrepancy, however, we should re-estimate the number of imTCs in the mouse MOB. We noticed that the widely-used markers for MOB projection neurons, such as TBR2, cholecystokinin, or Cdhr1, all label only sparse cells in the imTC-containing EPL zone, but much more abundant cells in the two flanking bands that contain MCs and eTCs (Fig. 2; Stahl et al., 2016), mirroring the distribution of birthdate-tagged OB subtypes in this study. Moreover, a recent study shows that a large fraction of neurons in the imTC-containing EPL zone are actually parvalbumin-containing inhibitory interneurons (Kato et al., 2013).

### Axon guidance of OB neurons with different birthdates

OB axons project through the LOT in a chronotopic manner (Inaki et al., 2004; Yamatani et al., 2004). Namely, within the tract, early growing axons occupy deeper regions while later growing axons are simply added to the ventral surface side of the preexisting axons. The present study confirmed that this temporal order of axon growth, in fact, reflects the birthdate order of the projection sources; the TM10.5-tagged neurons mainly consisting of AOB neurons projected the axons deep into the LOT (Fig. 4), whereas a more ventral surface space of the LOT were occupied by axons of late-born neurons including TM13.5-17.5 populations (Figs. 7–10).

This chronotopic axon organization within the LOT has been discussed as a potential consequence of physical competition among growing axons over open space on a first-come-first-served basis (Yamatani et al., 2004; Hirata and Iwai, 2019). A different form of guidance seemed to operate for the late-stage targeting of the axon terminals onto the aiCAP. The developmental processes observed here (Fig. 14) suggest that axons from the differently aged neurons do not compete for this target but are selectively guided to their specific targets, from the beginning, by distinct molecular signals.

### Neuronal birthdating based on the Neurog2 expression

The traditional birthdating method using thymidine analogs determines the timing of the final S phase in neuronal progenitors (Bayer and Altman, 1987), whereas the present birthdate tag makes use of the expression timing of the Neurog2 gene. Neurog2 is expressed in an oscillatory, fluctuating manner in neural progenitors and in a more sustained manner in neuronally committed cells (Shimojo et al., 2008; Imayoshi and Kageyama, 2014). The latter strong expression acts as the master determinant that directs neural progenitors into the neuronal lineage (Schuurmans and Guillemot, 2002; Imayoshi and Kageyama, 2014), and is likely to be exploited for Cre-loxP recombination in our birthdate tag approach. Indeed, the reported time course of Neurog2 expression in the G_1_/G_0_ phase after the final mitosis in cerebellar Purkinje cells (Florio et al., 2012) matches very well with the recombination time course observed in the present study (Fig. 1*C*).

Neuronal differentiation has been related to the last cell cycle (McConnell, 1989; McConnell and Kaznowski, 1991). More recent studies show that these events can actually be uncoupled experimentally, but are interconnected *in vivo* at various levels (Hardwick and Philpott, 2014). The key element of the relationship is that up-regulated neuronal differentiation genes such as Neurog2 put an end to the cell cycle, by regulating cell cycle components (Lacomme et al., 2012). There is however a clear deviation from this relationship. Basal progenitors in the cortex (Ochiai et al., 2009; Telley et al., 2016) are neuronally committed cells, but undergo one or two additional rounds of cell cycles to generate solely neurons. They express Neurog2, and in fact, this expression is supposed to be the critical inducer of these fate-restricted progenitors (Miyata et al., 2004; Britz et al., 2006). Thus, Neurog2 expression may be a metric more directly related to the timing of the neuronal commitment than the cell cycle exit.

Recently, new birthdating methods have revolutionized our understanding of neural development (Tabata and Nakajima, 2001; Saito, 2006; Govindan et al., 2018). For example, a new metric based on M phase-dependent cell positioning within the ventricular zone has yielded unexpected results about corticogenesis (Telley et al., 2016; Oberst et al., 2019). Likewise, different metrics of neuronal birthdating may highlight distinct biological consequences under certain situations.

### Future perspectives

Recent studies have revealed many more maps in the higher-order brain centers than previously expected, resulting from branched parallel pathways (Young, 1998; Laramée and Boire, 2014; Glasser et al., 2016; Tsukano et al., 2017). We thus wonder whether the detection of an olfactory map may be especially difficult, due to its uniqueness; the map does not represent real anatomic space, but rather repertoires of receptors, whose lack of spatial continuity makes it difficult to detect repetitive individual maps. Using an alternative approach, this study as well as a previous *Drosophila* study (Jefferis et al., 2001) demonstrate the effectiveness of anatomic dissection of projection neuron subtypes by birthdate-based classification. It is possible that this approach, in the future, will lead to the further subclassification of projection neuron subtypes and reveal additional maps that are buried in the seemingly random olfactory networks. Furthermore, the birthdate tag method will be useful for dissecting functional neuronal networks in other nervous systems as well.

## Synthesis

Reviewing Editor: Esther Stoeckli, University of Zurich Decisions are customarily a result of the Reviewing Editor and the peer reviewers coming together and discussing their recommendations until a consensus is reached. When revisions are invited, a fact-based synthesis statement explaining their decision and outlining what is needed to prepare a revision will be listed below. The following reviewer(s) agreed to reveal their identity: NONE.

Both reviewer acknowledge that the manuscript has been improved considerably and that critical points have been addressed in the revision. Despite the lack of statistical analysis of the results due to the small number of animals included in the study, we feel that the contribution to the field warrants publication of the study. The paper provides a new perspective on the central projections of olfactory bulb tufted cells and thus provides the basis for future functional studies on how olfactory information is processed. As so far no functional confirmation of the suggested pathways is available but would be required to put more emphasis on the study, we suggest to add a sentence in this direction to the Discussion. Something along the lines that this study provides the basis for future studies at the functional level that would confirm the anatomical observations reported in the manuscript.

## Author Response

Dear Dr. Esther Stoeckli,

We thank you and the reviewers for helpful comments and constructive feedback, and furthermore for the opportunity to resubmit a revised copy of this manuscript. According to all the comments, we have revised the manuscript thoroughly and responded to all the issues that you pointed out. Because this revision involves very extensive changes, only significant changes are indicated in blue colored fonts in the manuscript for the sake of clarity.

We think that the manuscript is now greatly improved, and hope that it is acceptable for publication in eNeuro.

Thank you for your generous consideration.

Here are point-to-point responses to your comments.

A major concern is the restricted labeling that is achieved by the method described in the paper. The labeled neurons represent only a subset of the population and it is not clear how representative these subpopulations are. In some cases, the number of cells that were labeled in the three animals used per group differ extremely. The labeled cells are poorly characterized.

We totally understand this concern. As you will see in the revised manuscript, we have completely changed the format of this manuscript to present axon projections of neuron populations tagged at different stages on a stage-by-stage basis. We also expanded our analyses to cover additional neuronal populations with other birthdates. We believe that these changes will provide a more accurate and clear picture about the neuron populations examined in this study. In addition, the labeled populations have been characterized in much more detail by additional experiments and datasets which will be explained one by one below.

Specific issues are:

Unfortunately, the conclusions are based on restricted labeling of mitral cells labeled at embryonic day 12.5 and tufted cells at embryonic day 15.5. While neurogenesis of both of these populations does occur on those days, it accounts for less than 50% of the total neurogenesis. Among mitral cells, neurogenesis is robust from ~E11 - E13.5, with a tail extending into E14/15. Tufted cells on the other hand are most robust from ~E14 - E17. Thus, it is not possible for the primary conclusion of this manuscript, the highly segregated projection of TCs to a small region of the tubercle and the MCs to the remaining area of the tubercle and throughout piriform cortex, to be valid until the full population and range of birthdates are studied.

In the revised manuscript, axon projections of neurons tagged at other stages have been provided. Specifically, the results for TM11.5 (Fig. 5, Lines 507-519), TM13.5 (Fig. 7, Lines 540-569) and TM17.5 (Fig. 10, Lines 610-620) populations are provided in addition to the original datasets of TM10.5 (Fig. 4), TM12.5 (Fig. 6) and TM15.5 (Fig. 8). Inclusion of all these data will provide a more complete representation of projection neuron subtypes examined in this study. Moreover, and more importantly, since we totally agree with this concern, we shifted our viewpoints from the comparison between MCs vs eTCs to the comparison of mixed neuron populations birthdate-tagged at different stages (e.g., Lines 11-13, 90-92, 102-105, 694-708). We think that this new format is more accurately matched with the results and conclusions of this study. Thus, while our conclusion may still be considered to be based on surveying a portion of all MCs and TCs, the more comprehensive data presented in the new manuscript have shown that the basic conclusion regarding existence of parallel projection pathways in the main olfactory system, remains relatively intact.

Many complex lines of mice are employed and the reason for using them is often not clearly stated. For example, if the G2A and Cdhr1 lines produce similar results with regard to axon labeling, why have both been used? A table clearly showing all of the lines and crosses would be very helpful.

We apologize for this confusion caused by our insufficient explanations. Because the usage of complex multiple mouse lines is inevitable in this study, descriptions about mouse lines and the reasons why they are required are greatly expanded in the revised manuscript (Lines 377-405, 459-491). In addition, a table of all mouse lines used is provided as Table1.

The authors do not explain well the use of a transiently expression to label cells. They state initially that they used only the Cre-ER but then crossed the neurogenin line to get better labeling. It is, in fact, the neurogenin's transient expression that is controlling their system, not the expression of the Cre-ER (see Zirlinger et al. (2002) PNAS 99: 8084 for a much clearer explanation.)

Again, we are sorry for our misleading descriptions about the birthdate tag line in the previous manuscript. To avoid such confusion, the name of our birthdate tag driver mouse line was changed to Neurog2CreER(G2A). In addition, we have explained more carefully the design of birthdate tag method (Lines 377-405). Furthermore, discussion about the usage of transient expression of Neurog2 for neuronal birthdating has been included in discussion (Lines 814-848).

There seems to be considerable variability in the number of cells labeled. Compare the numbers of labeled MCs and TCs in Figs. 1 - 3. Not even the ratios are constant (eg. Compare MC:TC in panels A and B of Fig. 3.

The numbers of neuron subtypes labeled are variable depending on which reporter gene is used. This is the main reason that we decided to use multiple reporter lines and compare their axon projections in order to obtain reliable results. Because previous descriptions about individual reporter lines were insufficient, in the revised manuscript, we created a new section to explain differences of reporter lines (Lines 459-491), and showed actual comparisons of axon projections in different reporter lines (Fig. 7, Lines 553-569, Fig.8 Lines 583-585).

Also, regarding quantification - “No statistical test was was performed in this study” (Methods, page 16). Not really acceptable if one is comparing ages, or regions, or subpopulations of cells, which they have done. One cannot presume to draw conclusions from cell counts in the absence of statistics

We should have explained our policy about statistical test more clearly. In the revised manuscript, we added explanation as to why we did not perform the statistical test (Lines 362-366). Here, we would like to point out three reasons. First, this study does not require any dichotomous judgement or does not involve any hypothesis testing. Development is a continuous process. I always find it inappropriate and misleading to apply a dichotomous significance testing in this continuous process (without a specific hypothesis). In this study, the purpose of quantification is to show a trend of the continuous developmental process but not to differentiate a specific developmental stage from the others. Second, according to this reviewer's comment, I did perform one-way ANOVA with post-hoc Tukey HSD test, the most common test for multiple comparison in biology, for datasets of Fig. 1D and Fig. 3A, C. The test results are just as expected (that is, only peak values have “statistical significances”). Therefore, it was very tempting for me to include these test results in the manuscript, but I would rather not to include them because I believe that the sample size (n=3) is not sufficient for such a testing. Although the ANOVA with post-hoc Tukey HSD test has been extensively used for analyses of small-sized data in many papers, I would like to make a decent decision regarding our study. Finally, eNeuro recently announced a policy about statistical analysis (https://www.eneuro.org/content/6/4/ENEURO.0259-19.2019). This editorial was really encouraging for me to depart from the obsession with the p-value-based testing. According to this editorial, I conducted “estimation statistics” for all our quantified data, but only the dataset of Fig. 1C turned out to have sufficient sample size for the bootstrap confidence estimation, and thus the analyzed result is included in the revised manuscript (Fig. 1C, Lines 364-366, Lines 1230-1237). In sum, I agree that dichotomous statistical testing is needed for some analyses, but I believe that inappropriate use of statistical testing is rather harmful in some situations.

Also, I note that correction factors were not used when doing the cell counts and that none of the bar graphs have standard errors.

Our previous descriptions may not be sufficiently clear. The numbers of neurons were, in fact, normalized by the tissue size (Fig. 1D, Fig. 3A, C). In the revised manuscript, we explained this point more clearly (Lines 291-294, 1239-1240, 1286-1287). Since we understand the difficulty related to these graphs, pie charts showing the overall proportion of neuron subtypes are added in the revised manuscript (Fig. 1G, Fig. 3 B, D, Lines 294-297, 422-423, 1250-1255, 1287-1292). These new presentations will be helpful to get a clear image of the birthdate-tagged mixed populations of neuron subtypes. Regarding the standard errors mentioned by this reviewer, because raw individual values are plotted in the graphs, we think that such average statistics are not needed. As explained in the statistical methods (Lines 358-362), this is generally considered as an appropriate way in presentation of small-sized data.

The projections of tufted cells in the anterolateral part of the tubercle is well known from the studies of Shin et al. (2010) which should be referenced. Similarly, the work of Walz et al. (J. Neurobio. (2006) 66: 835-846 should be discussed since they too used genetic markers to track axons in the piriform and tubercle.

The reviewer probably refers to the paper by Shin Nagayama et al (2010), which was discussed in the previous manuscript and so is in the revised manuscript. The report by Walz et al. (2006) is cited in the revised manuscript.

Page 15 - It would be helpful to know the stereotaxic coordinates that were used for injections.

We apologize for this mistake. The mice were head-fixed with a stereotaxic apparatus but injected with viruses according to our original coordinates, which are explained in the method (Lines 342-346).

The authors do not provide an independent determination of cell identity other than their location in the olfactory bulb, which may be confounded for those tufted cells proximal to the mitral cell layer.

We conducted subtype marker analyses of neurons tagged at different birthdates (Fig. 2C-F) and explained the results (Lines 439-457). The analysis involving these histochemical markers suggested that imTCs cells positioned proximal to the mitral cell layer are actually more sparse than previously described. We discussed this possibility together with other supporting data in the revised manuscript (Lines 782-793).

In Figures 5 and 6 the number of labeled mitral and tufted cells seems disproportionate in Panel A and J of Figure 5, for example, it appears all mitral cells are labeled following the 12.5 tamoxifen while only tufted cells on the medial wall of the olfactory bulb are labeled following tamoxifen at 15.5?

There seems a misunderstanding. At both TM12.5 and TM15.5, all the circumferential neurons (MCs and eTCs, respectively) are labeled. We are afraid that the arrows in the previous Fig. 6J may have led to this confusion. These arrows actually indicated the intrabulbar association fibers that are most prominent in the medial wall of the OB (Fig. 7A, 7I, 8A, 8G). In the revised manuscript, intrabulbar association fibers are explained in a more clear way in the main text and figure legends to avoid misunderstanding about these pictures (Lines 547-552, 580-582, 1248-1249, 1364-1365, 1392-1393).

Also in Figure 5, why isn't the LOT labeled in panels C, D, and E, and perhaps others. Even at high mag on the computer screen the labeling of the LOT is inconsistent with the heavy labeling of the cortex.

This is actually a very general issue about myelinated axons in the tract in immunostatining. The weaker fluorescence is probably caused by difficulty of penetration of antibodies into tightly bundled myelinated axons. Because this is very common not only in the LOT but also other tracts in many anatomical papers, we briefly mentioned it with a reference in the legend of Fig. 5 (1323-1324).

It seem premature to name as a nucleus the area receiving input from tufted cells born at embryonic day 15.5. It appears to be the lateral hook of the tubercle and may be developmentally innervated but not necessarily in a exclusive context.

According to this suggestion, we abandoned the name of the nucleus of external tufted cells, and instead use the name of this area (aiCAP: most anterolateral isolation of CAP compartments) in the revised manuscript (e.g. Lines 648-652). Also, in the revised manuscript, the spatial relationship of this target domain (aiCAP) and the lateral hook of the tubercle is more clearly described (Lines 596-599) and depicted in pictures (Figs. 5E', 6E', 7E', 7M', 8E', 8K', 9D).

1) Use of G2ACreER mice. Lines 108-114 and 355-359. The authors should explain better why this line is suitable for the experiments proposed. Why do the authors choose a “potential enhancer of neurogenin 2”? What are the “existing mouse lines that were not adequately efficient for application to stable birthdating analyses in the OB”? This comment is also applicable to the other lines used. For those who are not familiar with olfactory bulb circuitry, it is important to describe the context of the experimental design. I found particularly hard to understand lines 396-407.

We have completely rewritten these parts so as to clearly explain the answers to these questions in the manuscript (Lines 377-405, 459-491). Accordingly, descriptions become long, but we think that they make better senses.

2) Line 258. What does it mean “to reduce the litter effect”?

We apologize for this mistake; the “litter effect” should have been “litter bias”. In the revised manuscript. we explain more carefully what we would like to mean (Lines 270-273)

3) Methods section. Please include a table summarizing the lines used in the study, what are their main characteristics, references and neuronal populations labeled. The goal is having the table as a reference to follow more easily the results section.

We appreciate this helpful advice. The new Table1 that summarizes mouse lines (introduced at Line 128) should make it easier for readers to follow this study.

4) Line 185. Silvia Arber.

Lot of thanks. We corrected this awful mistake (Line 192).

5) Line 371 and Fig. 1D. I cannot see the dendritic morphology in the figure.

We added pictures showing dendrites in Fig. 1F. In addition, to show clear dendritic morphology, magnified black and white images of birthdate-tagged neurons are added in Figs. 5A', 6A', 7A', 7I', 8A', 8G', 10A'.

6) Fig 1E. Why do some nuclei appear labelled in the glomerular layer in TM17.5?

They are another subset of eTCs labeled at TM17.5. In the revised manuscript, we explain this subset in more detail (Lines 448-455, 610-620).

7) Fig. 2C-H. I would like to see a clearer image of the neuronal morphology of MCs and TCs. Lines 391-393 state that cell markers confirmed that TCs were labelled but I cannot see the dendritic tree of a TC.

To clearly show neuronal morphology of MCs and TCs, high-contrast black and white images of birthdate-tagged neurons are created and presented in Figs. 5A', 6A', 7A', 7I', 8A', 8G', 10A'.

8) Figures 7. It would be interesting to see the morphology of a neuron of the NeTC. It should be possible with TREtdTomato-sypGFP.

High magnification image of neurons in the target cell cluster of TM15.5 axons is provided in Fig. 7F (Lines 652-654). In this picture, we used an image immunostained for DARPP, because it shows the neuronal shapes most clearly.

9) Lines 494-497. I found the statement speculative. It can be moved to the discussion.

This speculative statement has been deleted.

10) Lines 567-573. The authors should clarify this point experimentally by providing images of the morphology of labelled TCs. The authors say “our preliminary observation...”. I cannot find the evidence in the experiments presented of the morphology described. I think this point should be addressed to clearly demonstrate the nature of the neurons labelled with the current method .

According to this constructive comment, we have added the analysis of TM17.5-tagged eTC population in the study (Fig.10). Morphological comparisons of the eTC subsets tagged at TM15.5 and TM17.5 are clearly shown in the revised figures (Fig. 8 A', G' Fig. 10A'), described in the result (Lines 573-582, 611-616), and further discussed in the discussion (Lines753-766).

- Remove the Abbreviations paragraph/list. Please only use nonstandard abbreviations if a term appears three or more times (the initial appearance of the term and one other time).

The abbreviation table was removed. Abbreviated names only appeared in figures are explained in the figure legends.

- The species studied is not mentioned in the abstract. Please make sure to update both the abstract in the article file and on the submission form.

The species “mouse” is mentioned in the abstract of the revised manuscript.
