# Supplementary figures and images for: A Novel Birthdate-Labeling Method Reveals Segregated Parallel Projections of Mitral and External Tufted Cells in the Main Olfactory System
Source: eNeuro. 2019 Nov 18;6(6):ENEURO.0234-19.2019. doi: 10.1523/ENEURO.0234-19.2019 (PMC6868177; doi:10.1523/ENEURO.0234-19.2019)

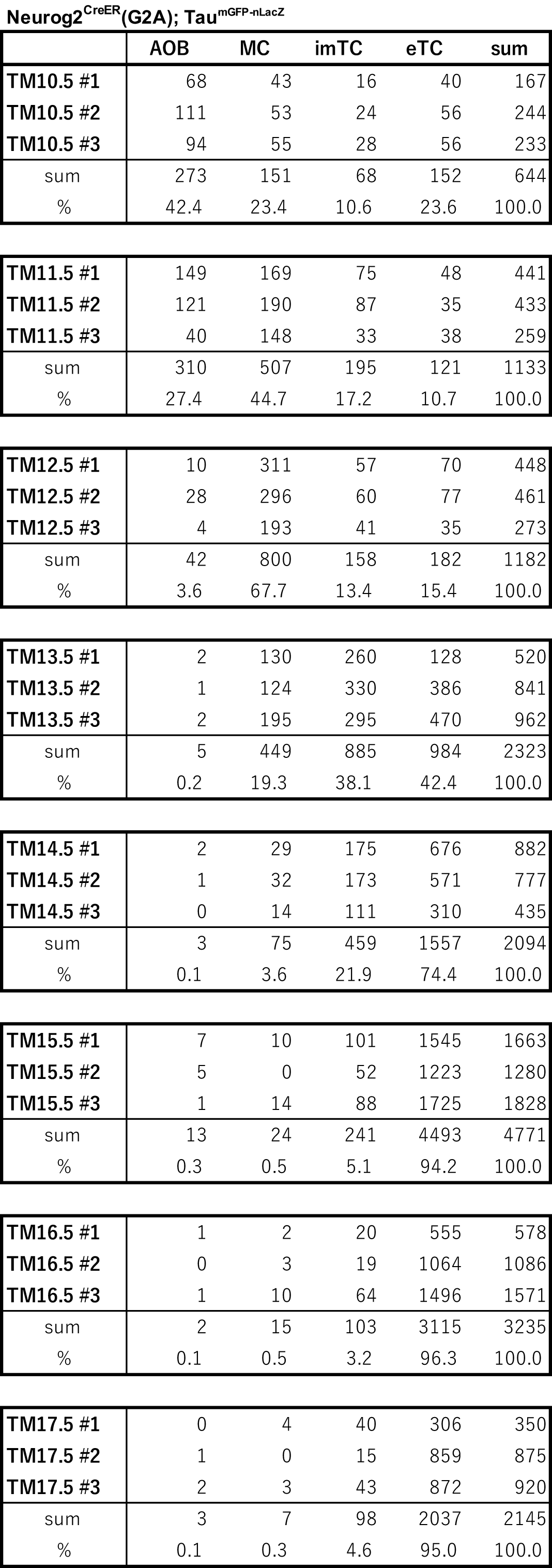

Supplement: Extended Data Figure 1-1 — Number of neurons counted in Fig.1. Download Figure 1-1, TIF file. [file sup_enu-eN-NWR-0234-19-s01.tif]

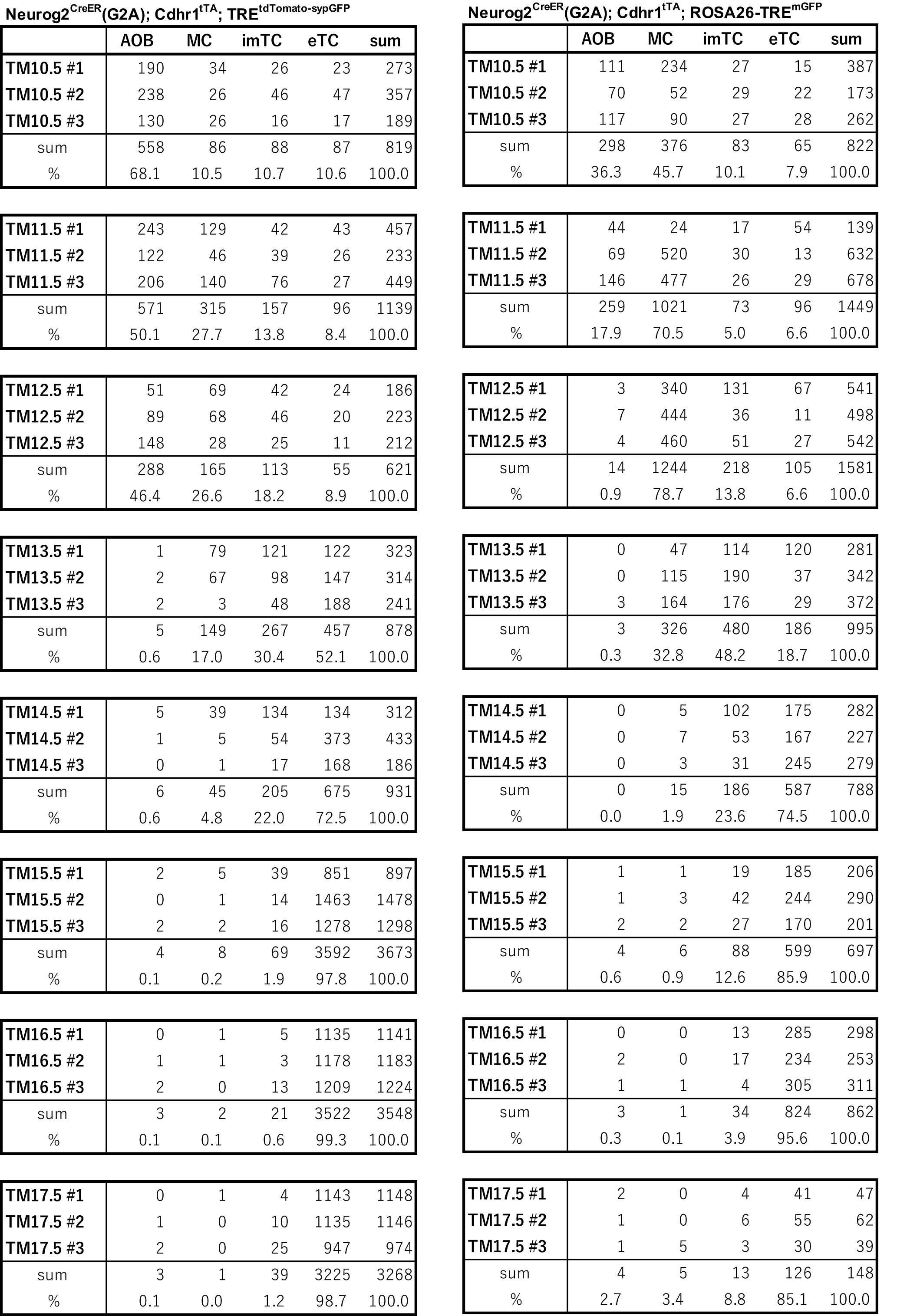

Supplement: Extended Data Figure 3-1 — Number of neurons counted in Fig.3. Download Figure 3-1, TIF file. [file sup_enu-eN-NWR-0234-19-s02.tif]
